# Prenatal Ethanol Exposure Misregulates Genes Involved in Iron Homeostasis Promoting a Maladaptation of Iron Dependent Hippocampal Synaptic Transmission and Plasticity

**DOI:** 10.3389/fphar.2019.01312

**Published:** 2019-11-07

**Authors:** Erwin De La Fuente-Ortega, Wladimir Plaza-Briceño, Sofía Vargas-Robert, Paola Haeger

**Affiliations:** Departamento de Ciencias Biomédicas, Facultad de Medicina, Universidad Católica del Norte, Coquimbo, Chile

**Keywords:** fetal alcohol syndrome, iron homeostasis, glutamatergic transmission, long term potentiation, adolescence, fetal brain programming

## Abstract

Prenatal ethanol exposure (PAE) induces behavioral maladptations in offspring, including a deficit in memory formation which is part of the umbrella sign of fetal alcohol spectrum disorder. Clinical and preclinical studies have shown that iron depletion exacerbates cognitive problems in offspring exposed to ethanol *in utero* and that PAE promotes dysregulation in brain iron homeostasis. However, the mechanisms underlying brain iron dysregulation and neuronal activity defects in adolescent offspring of PAE are unclear and poorly understand. Here, we used a PAE rat model to analyze messenger RNA (mRNA) and protein expression of iron homeostasis genes such as transferrin receptor (TfR), divalent metal transporter (DMT1), ferroportin (FPN1), and ferritin (FT) in brain areas associated with memory formation such as the prefrontal cortex (PFC), ventral tegmental area, and hippocampus. Interestingly, we found that 21 day old PAE rats have higher mRNA expression of DMT1 in the PFC, and TfR in the hippocampus, compared to control animals. In contrast FPN has lower mRNA expression in the PFC, and FT and FPN1 have lower expression in the hippocampus. In agreement with these results, we found a 1.5–2 fold increase of TfR and DMT1 protein levels both in the hippocampus and the PFC. Additionally, using an electrophysiological approach, we found that in hippocampal slices from PAE rats, iron treatment decreased long-term potentiation (LTP), but not AMPAR basal transmission (AMPAR fEPSP). In contrast, in control slices Fe-NTA did not affect LTP but decreased significantly the AMPAR fEPSP. Meanwhile, iron chelation with deferiprone decreased AMPAR transmission in PAE and control slices and decreased LTP only in controls slices. These results suggest that PAE affects iron homeostasis of specific brain areas—PFC and hippocampus—which could be involved in maladaptive cognition observed in this animal model.

## Introduction

The consumption of alcohol during pregnancy significantly alters fetal development and growth (Cuzon et al., 2008), and also affects cognitive processes throughout the child’s life (Ramsay, 2010; Skorput et al., 2015). Several clinical and preclinical studies have shown that prenatal ethanol exposure (PAE) can trigger cognitive disability and/or promote drug addiction during adolescence and adulthood (Bond and di Giusto, 1976; Phillips and Whitlock, 1976; Fabio et al., 2015) (Contreras et al., 2017). According to the World Health Organization, Chile has the highest alcohol consumption per capita within Latin America, with men consuming 13.9 L and women consuming 5.5 L of pure alcohol annually (Aros et al., 2009). It has been reported that out of 9,600 women interviewed in a study regarding prenatal exposure to ethanol, 57.4% admitted to have ingested alcohol at some point during pregnancy (Aros et al., 2009).

Interestingly, some studies using PAE mice models have shown that this condition triggers an imbalance in brain iron homeostasis (Miller et al., 1995; Carter et al., 2007; Huebner et al., 2016; Carter et al., 2017). However, these studies have focused on iron homeostasis in the whole brain during the gestational stage (Huebner et al., 2016), and in midbrain and subcortical areas during the postnatal (P17) stage of the offspring (Miller et al., 1995). However, it is unknown whether PAE affects iron homeostasis and/or neuronal activity in brain areas related with cognitive processes such as the prefrontal cortex (PFC) (Eichenbaum, 2017), the hippocampus (Burgess et al., 2002) and the ventral tegmental area (VTA) (Lisman and Grace, 2005) in adolescent animals.

Brain iron homeostasis plays important functions that require the expression of several genes which are important to maintain adequate intracellular iron levels. Iron is involved in fundamental processes such as cellular metabolism, cognitive processes, and memory formation (Aisen et al., 1999; Hidalgo et al., 2007; Carlson et al., 2009; Haeger et al., 2010). In the brain, iron is necessary for several steps involved in neurotransmitter production such as metabolic synthesis, packaging, uptake as well as degradation (Beard and Connor, 2003). In addition, iron contributes to synaptic plasticity through the catalytic production of reactive oxygen species (ROS) by the Fenton reaction; increasing intracellular calcium levels through ryanodine receptor activation (Hidalgo et al., 2007; Muñoz et al., 2011).

For neurons to comply with all their functions, they must maintain appropriate iron levels by regulating the expression of several iron homeostasis proteins which mediate ion uptake, storage, and release from neurons (Beard and Connor, 2003). Iron uptake is mediated by the TfR, which binds the iron (Fe^3+^)–transferrin complex at the cell surface and internalizes it towards endosomes for iron reduction (Fe^2+^), which then translocates to the cytosol by divalent metal transporter 1 (DMT1) (Núñez et al., 1990; Bogdan et al., 2016). DMT1 can also can be expressed at the cell surface allowing direct translocation of Fe^2+^ to the cytosol (Pelizzoni et al., 2012). Once in the cytosol, Fe^2+^ can be stored by the cytosolic protein FT, used by mitochondria, or constitute a free or labile iron fraction (Kruszewski, 2003; Valko et al., 2005; Mackenzie et al., 2008). Excess of intracellular Fe^2+^ is exported from the neurons through ferroportin 1 (FPN) (Wu et al., 2004; Boserup et al., 2011). In response to iron level fluctuation, neurons can regulate the expression of genes involved in iron homeostasis (e.g. TfR, DMT1, FPN, or FT) mainly by a posttranscriptional mechanism involving the iron-response element (IRE)/iron regulatory protein (IRP) system (Wang and Pantopoulos, 2011; Singh et al., 2014). Moreover, the systemic iron metabolism can be regulated by hepcidin (gene HAMP), an hormone released by liver that regulate iron by interacting with its receptor FPN (Collins et al., 2008). HAMP also can be expressed by glia in the brain (Urrutia et al., 2013).

The effect of PAE together with iron depletion or a supplementation diet have been studied mainly during gestation until G20 or until the early postnatal (P14/P30) development of rats by assessing the expression of some brain iron homeostasis proteins, and morphological alterations (apoptosis and myelination) (Rufer et al., 2012; Huebner et al., 2016). Particularly, it has been described that PAE reduced postnatal iron levels and increases the expression of FT and transferrin (Tf) at whole cerebral cortex (Miller et al., 1995), meanwhile iron deficient PAE rats exacerbate the reduction of iron levels at gestational day 20–10 in total brain and reduce the expression of iron homeostasis proteins (e.g. Tf/TfR, and FT) without changes to others (e.g. FPN and DMT1) (Rufer et al., 2012; Huebner et al., 2016). However, these studies have not evaluated the effects of PAE on the hippocampus and neuronal activity of adolescent offspring.

How PAE could affect iron homeostasis genes in specific brain areas related with cognitive behaviors in adolescent offspring and how iron fluctuations can affect neuronal activity were the main aims of this study. To this end, we used a PAE rat model to analyze the messenger RNA (mRNA) and protein expression of iron homeostasis genes (TfR, DMT1, FPN1, and FT) at three different brain areas associated with cognitive impairment of FASD: the PFC, the VTA, and hippocampus. Interestingly, we found that P21 rats with PAE presented increased expression of TFR in the PFC, and DMT1 in the hippocampus, meanwhile FPN in the PFC, FT and FPN in the hippocampus, decreased their mRNA expression. In agreement with this, we found a 1.5–2 fold increase of TfR and DMT1 protein levels in the PFC and hippocampus by Western-blot. Additionally, using an electrophysiological approach on hippocampal slices of P21 rats, we found that iron supplementation decreased long-term potentiation (LTP), but not the AMPAR synaptic transmission, of PAE rats compared to control slices. Meanwhile, iron chelation with deferiprone (DFP) produced a significant decrease of LTP in controls rats, but not in PAE rats. These results suggest that PAE affects the iron homeostasis in specific brain areas associated with memory and learning, the PFC and the hippocampus. These alterations may underlie the maladaptive behavior observed in the PAE animal model.

## Material and Methods

### Animals and Prenatal Alcohol Exposure Treatment

We followed the protocol described previously by Contreras et al., 2017. Pregnant Sprague–Dawley rats were exposed to ethanol (10% v/v) and 64 mg/l of sucralose (daily). Ethanol consumption was initiated on day 5 ± 2 days of gestation until 1 week after the offspring were born (P7). The consumption of liquid and food was monitored during the consumption period. After the ethanol protocol, rats were left with food and water *ad libitum*. Offspring were weaned at 21 days after birth (P21) and separated by sex. Protocols for rat handling were carried out in accordance with the recommendations of the Assessor Committee in Bioethical guidelines from the National Fund for Scientific and Technological Development (FONDECYT, Chile) and approved by the Bioethic, Scientific, and Animal Care and Use Committee of the Universidad Católica del Norte, Chile.

### Brain Sample Extraction

Different brain samples were extracted by microdissection. The brain region enriched of PFC and VTA was extracted from limited slices approximately from 4.2 to 2.7 mm and −5.2 to −6.8 mm from bregma, respectively, according to the Paxinos and Watson (1998). From the same brain, whole hippocampus was also removed. Samples were extracted from P21 and P70–78 offspring, which were exposed or not to ethanol in utero. Samples were collected on dry ice and stored at −80°C until processed.

### Tissue Homogenization and Western-Blot

We used the protocol described by Contreras (Contreras et al., 2017). Briefly, 100 mg of brain tissues (PFC, hippocampus, or VTA) were homogenized with 300 µl lysis buffer (20 mM MOPS/Tris pH 7, 0.3 M sucrose, 2 mM EDTA, 2 mM EGTA, 1% NP-40, and 0.1% sodium dodecyl sulfate), plus protease inhibitors. 10–30 µg of proteins was suspended in 3X loading buffer and denatured for 10 min at 70–80°C. Proteins were separated by 10% sodium dodecyl sulfate polyacrylamide gel electrophoresis (Minigel-BioRad), and transferred to 0.2 µm nitrocellulose membranes (Whatman-Protran #10401396, Merck). The membranes were blocked with 5% nonfat milk in phosphate-buffered saline (PBS) for 1 h at room temperature and incubated for 18 h at 4°C with the following primary antibodies, anti-β-actin (Sigma-Aldrich, 1:10,000), anti-TfR (Thermofisher Scientific, H68.4, 1:500), anti-DMT1 (Gift from Dr Marco Tulio Nuñez, 1:1,000 (Haeger et al., 2010). The membranes were washed three times with 0.2% Tween 20 in PBS at room temperature and incubated with secondary antibodies conjugated to horseradish peroxidase (HRP) (antirabbit HRP or antimouse HRP, both from Cell Signaling Tech. Danvers, USA) dissolved in 3% albumin-PBS 1X for 1 h at room temperature. After washing the blots three times with 0.2% Tween-PBS, they were exposed to chemiluminescent substrates (p-coumaric acid/luminol) for 1 min and scanned with a C-digit blot scanner (LI-COR). The bands were quantified with the ImageJ software (National Institutes of Health, USA) and normalized to β-actin.

### Quantitative Reverse Transcription PCR

**Figure 1:**
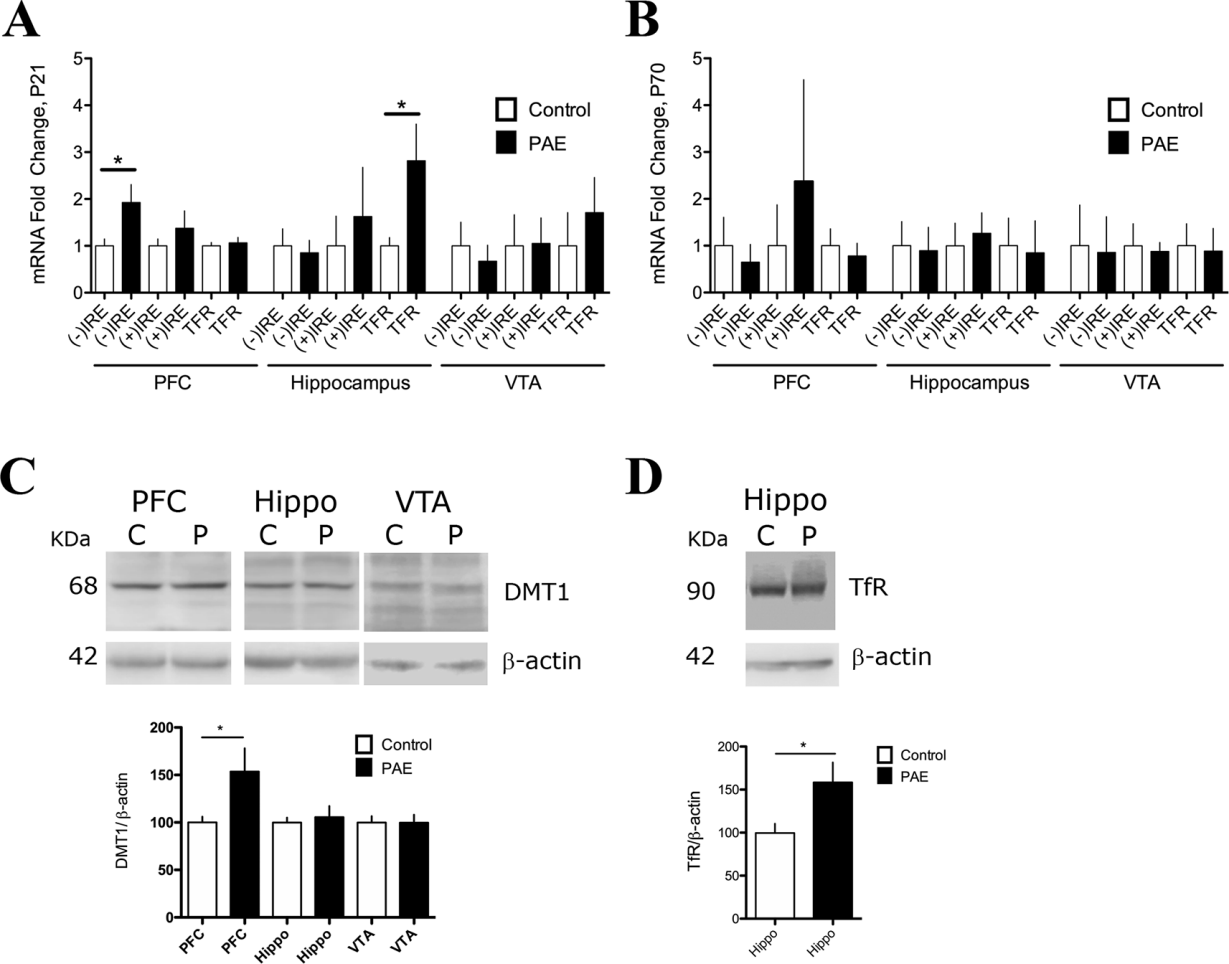
Prenatal ethanol exposure (PAE) affected messenger RNA (mRNA) and protein expression of iron transporters divalent metal transporter (DMT1) and transferrin receptor (TfR) in rat brain. **(A)** Relative mRNA expression of DMT1 isoforms [(−) IRE and (+) IRE], and TFR in the prefrontal cortex (PFC), hippocampus, and ventral tegmental area (VTA) of P21 PAE rats compared to respective brain areas of P21 controls rats. Graph A shows mRNA expression (fold change) in controls and PAE calculated using formula 1 and 2 (*Material and methods*) with means ± SEM. *p < 0.05 for comparison by Mann–Whitney. N 5. **(B)** Relative mRNA expression of the same genes and brain areas of PAE and control rats as A, but for ages P70–78. Graph B shows mRNA expression (fold) in controls and PAE, calculated using formula 1 and 2 (*Material and methods*) with means ± SEM. *p < 0.05 for comparison by Mann–Whitney. N 5 litters. **(C)** Western blot analysis of DMT1 expression in control (C) and PAE (P) P70–78 rats in the PFC, hippocampus (Hippo), and VTA. N = 10 litters. **(D)** Western blot analysis of TfR expression in control (C) and PAE (P) of P21. N = 6 litters. DMT1 and TfR proteins were immuno-detected using specific antibodies and normalized to β-actin (42 kDa). Graphs in C and D show means ± SEM, and *p < 0.05 for the indicated comparisons by Mann–Whitney test.

**Figure 2:**
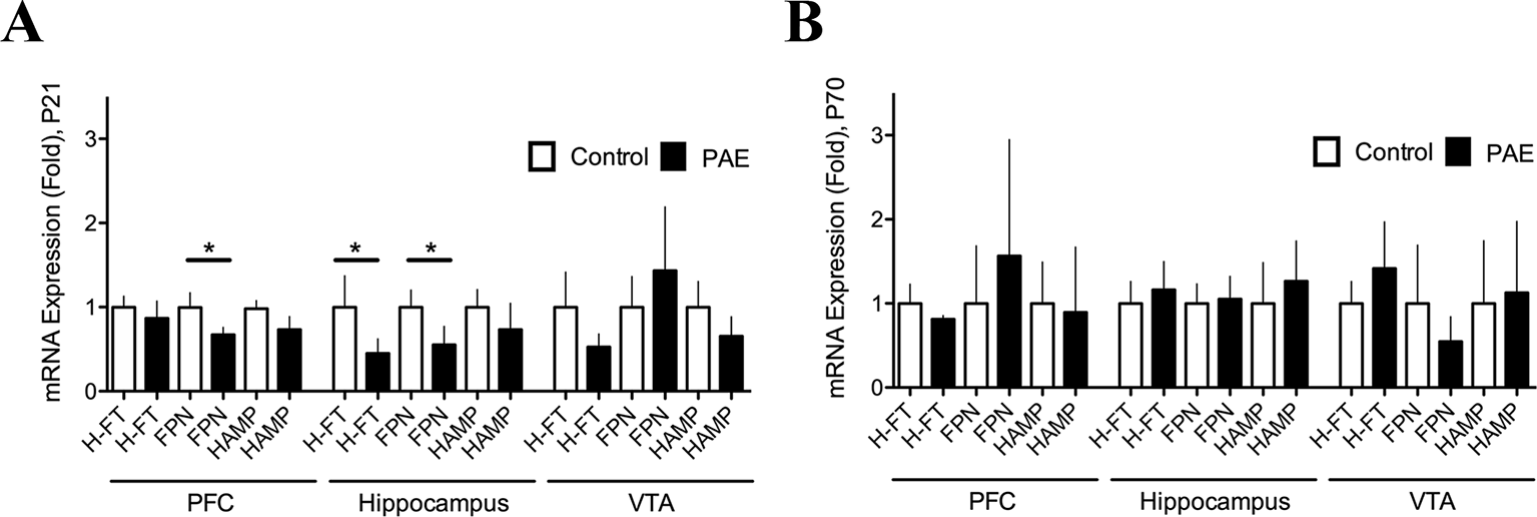
Prenatal ethanol exposure (PAE) affected ferritin and ferroportin messenger RNA (mRNA) expression in rat brain. **(A)** Relative mRNA expression of ferritin (H-FT), ferroportin (FPN), and hepcidin (HAMP) in brain areas of P21 PAE rats—prefrontal cortex (PFC), hippocampus, and ventral tegmental area (VTA)—compared to respective brain areas in control rats at P21 days old. **(B)** Relative mRNA expression of the same genes and brain areas of PAE rats as A, but for day P70–78 compared to the respective brain areas in P70–78 control rats. Graphs A and B show mRNA expression (fold change) in controls and PAE calculated using formulas 1 and 2 (*Material and methods*) with means ± SEM. *p < 0.05 for comparison by Mann–Whitney. N 8 litters.

We performed quantitative reverse transcription PCR (RT-qPCR) analysis for DMT1-1B (+IRE and −IRE isoforms), TfR, FT, FPN, and HAMP in the PFC, hippocampus, and VTA of PAE rats, comparing them with controls using the method previously described by Contreras 2017 (Contreras et al., 2017). Brain tissues samples (50–100 mg) were homogenized with TRIzol (TRIzol^R^ Reagent, Invitrogen ™ Life Technologies, USA) to extract total RNA, treated with DNase (1 U, Turbo DNA-free™ Kit, Life-Technologies) to eliminate contaminant DNA. First strand complementary DNA was synthesized with the Improm II ™ kit (Promega, USA), the reaction tube contained 10 µg RNA, 3 mM MgCl_2_, 0.5 mM dNTPs, reaction buffer [50 mM Tris–HCl (pH 8.3 at 25°C), 75 mM KCl, and 10 mM DTT], 20 U (1 µl) of reverse transcriptase (Improm II TM, Promega, USA), and nuclease-free water, reaching 5 µl of final reaction volume. For qPCR, specific primers for genes involved in iron homeostasis and housekeeping genes (β-actin or GAPDH) with melting temperatures (Tm) of 60°C and amplicons of approximately 100–200 bp are shown in **Table 1**. The qPCR reaction contained 5 µl of 2X SYBR green master mix (kapa sybr^®^fast, biosystems, USA), complementary DNA (5 µl), 50 nM of each primer, and nuclease-free water until the final reaction volume reached 10 µl. Real-time PCR reactions were run with the Applied Biosystems StepOne^™^ system (Applied Biosystems) using the following amplification conditions: initial denaturation for 10 min at 95°C followed by 40 cycles of denaturation at 95°C for 15 s, and annealing/extension at 60°C for 30 s. Gene expression levels were normalized to housekeeping genes, β-actin or GAPDH, according to the PCR efficiency similarity. To determine differences between the expression of iron homeostasis gene (fold changes) in the PAE rat groups and the control group (**Figures 1** and **2**, and **Supplementary Tables 2** and **3**), the expression of iron homeostasis genes were quantified in both groups using 2−ΔCt as previously described (Schmittgen and Livak, 2008). Briefly, the mRNA expression of iron homeostasis genes in each group was calculated:

$$\begin{array}{l} \mathrm{Formula}\;1,\;\mathrm{mRNA}\;\mathrm{expression}\;\mathrm{of}\;\mathrm{control}\;\mathrm{group}\;=\\ 2^{-∆\mathrm{Ct},\mathrm{control}}/2^{-∆\mathrm{Ct},\mathrm{Mean}\;\mathrm{control}} \end{array}$$

$$\begin{array}{l} \mathrm{Where},\;∆\mathrm{Ct},\;\mathrm{control}\;=\\ \left(\mathrm{Ct}\;\mathrm{gene}\;\mathrm{of}\;\mathrm{interest}\;-\;\mathrm{Ct}\;\mathrm{internal}\;\mathrm{housekeeping}\right)\;\mathrm{control} \end{array}$$

$$\mathrm{and}\;2^{-∆\mathrm{Ct},\;\mathrm{Mean}\;\mathrm{control}}=\;(\sum \;2^{-∆\mathrm{Ct},\;\mathrm{control}})/n\;\mathrm{litters}$$

$$\begin{array}{l} \mathrm{Formula}\;2,\;\mathrm{mRNA}\;\mathrm{expression}\;\mathrm{of}\;\mathrm{PAE}\;\mathrm{group}\;=\;\\ 2^{-∆\mathrm{Ct},\;\mathrm{PAE}}/2^{-∆\mathrm{Ct},\;\mathrm{Mean}\;\mathrm{control}} \end{array}$$

$$\begin{array}{l} \mathrm{Where},\;∆\mathrm{Ct},\;\mathrm{PAE}\;=\\ \left(\mathrm{Ct}\;\mathrm{gene}\;\mathrm{of}\;\mathrm{interest}\;-\;\mathrm{Ct}\;\mathrm{internal}\;\mathrm{housekeeping}\right)\;\mathrm{PAE} \end{array}$$

$$\mathrm{and}\;2^{-∆\mathrm{Ct},\;\mathrm{Mean}\;\mathrm{control}}=\;(\sum \;2^{-∆\mathrm{Ct},\;\mathrm{control}})/n\;\mathrm{litters}$$

**Table 1:** Nucleotide sequences of the forward (F) and reverse (R) primers used for qRT-PCR of genes involved in iron homeostasis and housekeeping genes in *Rattus norvegicus*.

Gene	Sequences (5’-3’)	Amplicon (bp)	Reference
ratDMT1 (+) IRE	F: GCCTGTCTGTCTGTCTTTGC	134	(Pelizzoni et al., 2012)
	R: CCCAGTGTTTCCCAACTAACA		
ratDMT1 (-) IRE	F: AAGGCGAAGAAAGATCTGGAG	113	(Andersen et al., 2007)
	R: CCACAGGCCGCTGTTTG		
ratTFR1	F: ATACGTTCCCCGTTGTTGAGG	111	(Naz et al., 2012)
	R: GGCGGAAACTGAGTATGGTTGA		
ratFPN1	F: TCGGTTCCTCTCACTCCTGT	198	(Urrutia et al., 2013)
	R: GTGGAGAGAGAGTGGCCAAG		
ratHAMP	F: GAAGGCAAGATGGCACTAAGCA	102	(Moriconi et al., 2009)
	R: TCTCGTCTGTTGCCGGAGATAG		
ratBeta-actin	R: TCTACAATGAGCTGCGTGTG	130	(Haeger et al., 2010)
	R: TACATGGCTGGGGTGTTGAA		
ratGAPDH	F: AACGACCCCTTCATTGAC	191	(Dong et al., 2010)
	R: TCCACGACATACTCAGCAC		

In addition, to determine the relative mRNA levels between PAE and control animals were calculated using the 2^−ΔΔCt^ method, where ΔΔCt = (Ct gene of interest − Ct internal housekeeping)_PAE_ − (Ct gene of interest − Ct internal housekeeping)_Control_ (Livak and Schmittgen, 2001; Pfaffl, 2001). At least three animals from each litter were analyzed and values were averaged per group, a minimum of three independent litters were analyzed.

### Hippocampal Slice Electrophysiology

We followed the protocol described by Nguyen and Kandel, 1997. Acute transverse hippocampal slices with a thickness of 400 mm were prepared from 17 to 30 days old Sprague–Dawley rats using a DTK-1000 Microslicer (Ted Pella, Inc.) in ice cold dissection buffer (in mM: 215 sucrose, 2.5 KCl, 1.6 NaH_2_PO_4_, 26 NaHCO_3_, 4 MgSO_4_ × 7H_2_O, 4 MgCl_2_, 1 CaCl_2_, and 20 glucose, bubbled with a mixture of 5% CO_2_ and 95% O_2_). Slices were incubated for 30 min to 1 h at room temperature in artificial cerebrospinal fluid (ACSF, in mM: 124 NaCl, 2.5 KCl, 26 NaHCO_3_, 1 NaH_2_PO_4_, 2.5 CaCl_2_, 1.3 MgSO_4_, and 10 glucose, bubbled with a mixture of 5% CO_2_ and 95% O_2_). Hippocampal slices were visualized using a Nikon Eclipse FN1 microscope. All experiments were performed at 28 ± 1°C in a submersion-type recording chamber perfused at 2 ml/min with ACSF supplemented with the GABAA receptor antagonist picrotoxin (PTX 100 µΜ). fEPSPs were evoked by stimulating Schaffer collaterals with a glass microelectrode (3–4 MΩ, filled with NaCl 1 M) positioned in the stratum radiatum of the CA1 area at 100 µm further than recording electrode located in the same region. Stimulation intensity was adjusted to elicit fEPSP amplitudes that were around 40% of the maximum size. For basal AMPAR transmission analysis, after 10–15 min of a stable baseline, slices from control or PAE rats were incubated with 20 µM Fe-NTA (a donor of Fe^3+^ as the complex FeCl_3_–sodium nitrilotriacetate, Fe-NTA, 1:2.2, mol:mol), 100 µM DFP, or saline solution (vehicle) in an ACSF buffer. Slices that showed maximal fEPSPs >2 mV were rejected. Then basal transmission was measured for 40–45 min compared to the baseline average.

Paired-pulse ratio (PPR) was calculated by delivering two pulses at 100-ms inter stimulus interval and was defined as the ratio of the amplitude of the second fEPSP to the amplitude of the first fEPSP (fEPSP2/fEPSP1). Fiber Volley (Fv) amplitude was calculated using the peak, and amplitude of the descending phase of the FV curve was measured by recording the magnitude of the fEPSP as a function of stimulus intensity.

For LTP analysis, slices from control or PAE rats were preincubated with 20 µM Fe-NTA, 100 µM DFP, or vehicle (saline solution) in ACSF during 40 min and were continually perfused during the experiment. Plasticity was induced by high frequency stimulation (HFS) (100 Hz, 1 s). In all experiments the difference and comparison between the averaged-baseline and the responses after the induction was determined. All recordings were performed using a MultiClamp 700B amplifier (Molecular Devices), elicited at 15–20 s intervals, filtered at 2.4 kHz, and acquired at 10 kHz, using custom-made software written in Igor Pro 6.36 (Wavemetrics). Unless otherwise indicated, all electrophysiological values are provided as mean ± SEM and illustrated traces are the average of 30–40 responses.

All the experiments were repeated at least three times with different litters. For electrophysiological recordings, three to five acute hippocampal slices per subject were used and pooled to count as one litter.

### Statistical Analysis

Data from groups, control and PAE, are presented as means ± SEM. For relative mRNA expression of iron homeostasis genes between PAE group and controls (**Figures 1A, B** and **Figures 2A, B**), the means ± SEM were analyzed by nonparametric Mann–Whitney. The comparison of mRNA expression between brain areas—PFC, hippocampus, and VTA—at P21 or P70–78 (in **Supplementary Figure 1**, and **Supplementary Table 1**), was analyzed by nonparametric Kruskal–Wallis, Dunn’s multiple comparison test. The comparison of mRNA expression between different ages, P21 and P70–78, at specific areas was analyzed by Mann–Whitney test. For relative mRNA expression of genes involved in iron homeostasis between different ages (P21, P70–70) (**Supplementary Figure 2**), was calculated using the 2^−ΔΔCT^ and statistical analysis by one-sample t-test, indicating significance (*p < 0.05, **p < 0.05). For protein expression the differences in mean values between two conditions were compared by a Mann–Whitney test. The electrophysiological data were analysed with two way ANOVA repeated measures, followed by Tukey’s *post-hoc*. In some particular cases, we also conducted planned comparation to analyze the effect of iron before and after treatment (paired t test) or iron effect of PAE LTP (unpaired t test). The partial eta squared (η²p) or Cohen’s d were used to inform effect size and the alpha level was kept at 0.05 across tests. Was calculated using the following formula: sum of square (SS)/(SS + error of SS).

## Results

### PAE Altered the Expression of Iron Homeostasis Genes in Brain Regions Involved in Learning and Memory Processes

Brain iron homeostasis is a complex processes modulated by several proteins which are expressed in the brain, including DMT1 isoforms, TfR, FT, FPN, and the HAMP (Hentze et al., 2004; Ke et al., 2005; Singh et al., 2014). DMT1-1B isoforms (+IRE and −IRE) are expressed exclusively in the brain (Ke et al., 2005). First we evaluated the basal expression of these genes in control littermates. We evaluated gene expression in different brain regions (PFC, hippocampus, and VTA) and ages (P21 respect to P70–78) by RT-PCR and normalized to β-actin (2^−ΔCT)^ (**Supplementary Figures 1A–F**, and **Supplementary Table 1A**). The analysis showed that some genes presented significant differences between the PFC, hippocampus, and VTA at P21 or P70–78 (using nonparametric Kruskal–Wallis test following Dunn’s multiple comparison test, **Supplementary Table 1A**). At P21 the isoform DMT1 (−) IRE and (+) IRE are expressed at higher levels in the VTA compared to the hippocampus (*p* = 0.0009, *p* = 0.0005, respectively); HAMP mRNA are expressed in higher levels in the VTA compared to the hippocampus (*p* = 0.00001); FPN are expressed in higher levels in the hippocampus compared to the PFC (*p* = 0.0235). While at P70–78 we did not find significant changes in iron homeostasis genes between the brain areas (**Supplementary Figure 1**). In addition, we analyzed whether iron homeostasis genes in specific brain areas present differences in expression levels between ages P21 and P70–78 (**Supplementary Table 1B**). We found that FPN expression decreased in the hippocampus in P70 compared to P21 (*p* = 0.0159, Mann–Whitney test). Meanwhile, HAMP mRNA expression increased in the PFC in P70 compared to P21 (*p* = 0.00375 Mann–Whitney test).

Next, we evaluated the effects of PAE on DMT1 and TFR gene expression at the mRNA and protein levels (**Figures 1A–C**). Using RT-qPCR and (2^−ΔCt^ ^PAE^/2^−ΔCt^ ^Control^, formula 1 and 2, *Material and Methods*), we found that the DMT1 (−) IRE mRNA isoform was increased by 1.925 ± 0.38 fold in the PFC (*p* = 0.0415, Mann–Whitney test), but it was not affected in the hippocampus and VTA of P21 PAE rats (**Figure 1A**, **Supplementary Table 2A**). In addition, P70–78 PAE rats did not present significant difference in DMT1 mRNA isoforms in P70–78 rats in these three areas analyzed (**Figure 1B**, **Supplementary Table 2B**). The comparative expression of these genes in different ages and brain regions can be observed in **Supplementary Figure 2A**. Consistent with the qRT-PCR analysis, we found that the PFC of PAE rats, but not hippocampus and VTA, presented a significant increased expression of DMT1 isoform proteins (bands of 68 kDa) immuno-detected with an antibody against the N-terminal domain (**Figure 1C**) (PAE 153.5 ± 24.54 N = 6, vs. controls 100 ± 6.05 N = 6, p = 0.0315, Mann–Whitney test). Additionally, we found that adolescent P21 PAE rats presented a significant increase in TFR mRNA (2.818 ± 0.7804 N = 9, p = 0.0071, Mann–Whitney test) in the hippocampus, but not in the PFC and VTA, compared to P21 control rats (**Figure 1A**, **Supplementary Table 2A**). Meanwhile in P70–78 PAE rats, TFR mRNA expression was unaffected (**Figure 1B**, **Supplementary Table 2B**). Consistent with the high expression of TFR mRNA in the hippocampus, Western blot analysis confirmed a significant increase of TfR protein expression (PAE 158 ± 23.17 versus controls 99.63 ± 10.80, p = 0.0043, Mann–Whitney test) at the hippocampus in P21 PAE rats compared to control rats (**Figure 1D**). Altogether, these results indicated that prenatal ethanol exposure changed the expression of proteins involved in iron uptake in a tissue-selective manner in P21 rats. Overexpression of the DMT-1B/(−)IRE isoform occurred in the PFC, meanwhile TfR overexpression was observed in the hippocampal region, and no changes occurred in the VTA of P21 PAE rats. Furthermore, cellular misregulation of DMT1 and TFR also suggest that additional genes involved in iron homeostasis can be misregulated in PAE rats.

### PAE Altered the Expression of the Cellular Iron Storage Protein (Ferritin) and Iron Release Protein (Ferroportin) in Brain Regions Involved in Learning and Memory Processes

**Figure 3:**
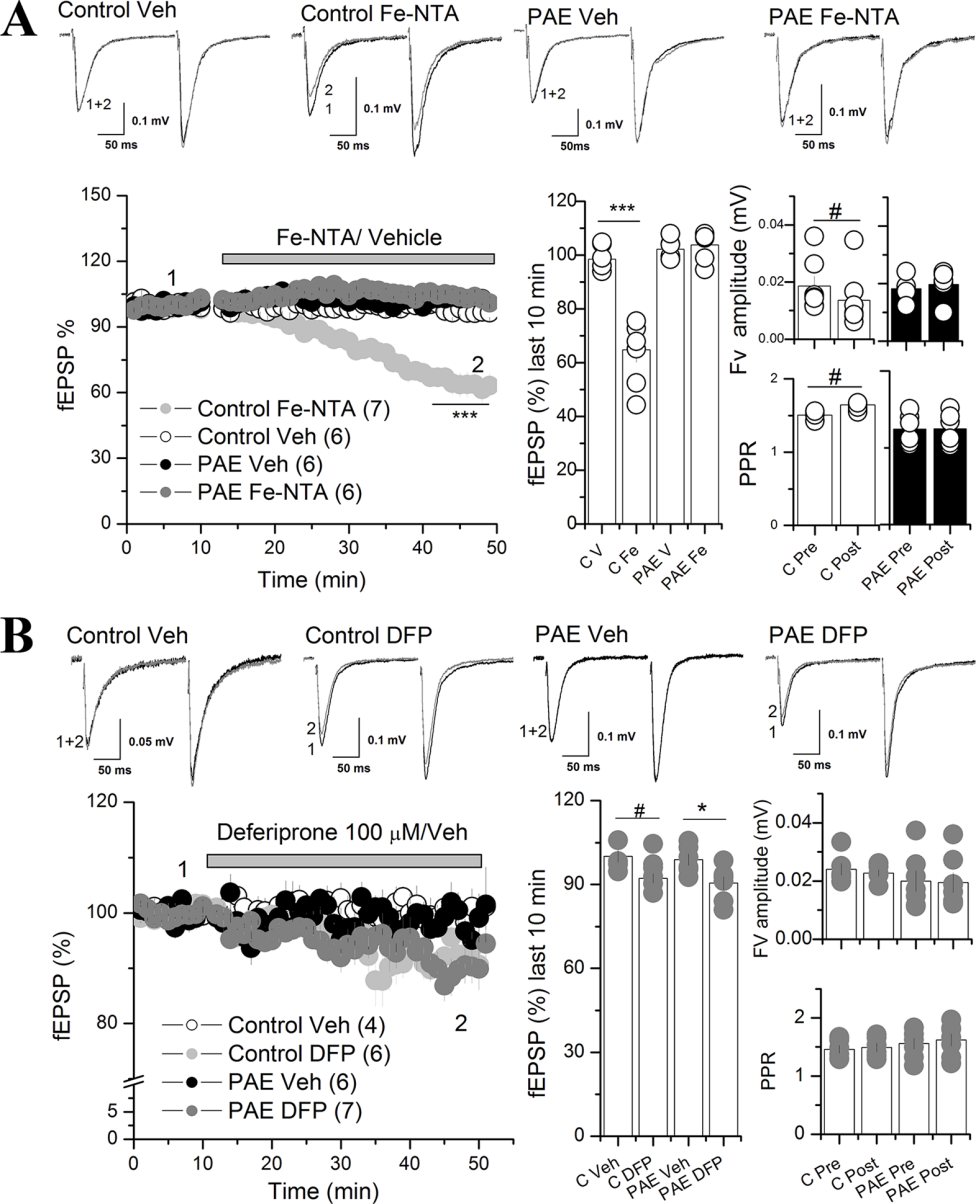
Iron addition (Fe-NTA) and iron chelator (DFP) affects AMPAR synaptic transmission at the CA1 of the hippocampus of PAE rats compared to control rats. **(A)** Effect of Fe-NTA on basal transmission of PAE and control rats. Basal transmission (% fEPSP) was recorded on slices from control and PAE rats and 20 μM Fe-NTA or vehicle (Veh, V) was bath applied as is indicated in the graph. Bar graphs represent the average % fEPSP during the last 10 min, the fiber volley (Fv) amplitude and PPR pre HFS (Pre) and last 10 min of recording (Post) was represented in bar graphs. Note that FeNTA reduced basal transmission in control but not in PAE rats. Fv was decreased meanwhile PPR was larger (fEPSP2/fEPSP1) in control rats and not in PAE. **(B)** The effect of deferiprone (DFP) on basal transmission of PAE and control rats. After baseline, 100 μM DFP or vehicle (Veh, V) was bath applied as is indicated in the graph. Note that DFP incubation reduced basal transmission in PAE rats and controls. Fiber volley (Fv) amplitude and paired-pulse ratio (PPR) were not affected in PAE or control rats. In all panels, sample traces were taken at the times indicated by numbers on the summary plot; the number of litter is indicated in parentheses. ***p < value 0.0001, *p < value 0.05, repeated measured two way ANOVA followed Tukey’s *post-hoc* test. ^#^p < 0.01 paired t test **(A)** and unpaired t test **(B)**.

In order to test whether additional genes involved in iron homeostasis were altered by PAE we analyzed relative mRNA expression of FT and/or FPN by RT-qPCR (**Figures 2A, B**, **Supplementary Tables 3A, B**). We found that P21 PAE rats presented a significantly decreased expression of FPN mRNA (PAE 0.6738 ± 0.08506 N = 8 versus control 0.9963 ± 0.1759 N = 8, p = 0.0415, Mann–Whitney test) in the PFC, and both the heavy chain-ferritin (H-FT) (PAE rats 0.4513 ± 0.1710 N = 9 versus control 0.9997 ± 0.3728 N = 9, p = 0.0122, Mann–Whitney test) and FPN mRNA (PAE rats 0.5541 ± 0.2172, N = 8, versus control rats 0.9996 ± 0.2025 N = 8, p = 0.019, Mann–Whitney test) were significantly decreased in the hippocampus compared to P21 control rats (**Figure 2**, **Supplementary Table 3A**). While P70–80 PAE and control rats did not present significant changes in mRNA expression of these genes (**Figure 3B**, **Supplementary Table 3B**). In addition, we analyzed gene expression of HAMP, a hormone that regulates iron homeostasis by inflammatory stimuli in the brain (Urrutia et al., 2013), however we did not find significant variation of HAMP mRNA in PAE rats both at P21 and P70–78 (**Figure 2A**, **Supplementary Tables 3A, B**). The comparative expression of these genes at different ages and brain regions can be observed in **Supplementary Figure 2B**. Thus, these results show that PAE altered the expression of genes involved in cellular iron storage (FT) and iron release (FPN) in a tissue specific manner and in an inverse manner than that of DMT1 and TFR. While in PAE rats the H-FT and FPN genes were downregulated, the iron homeostasis genes involved in iron-uptake such as DMT1 and TfR were upregulated in brain areas involved with learning and memory process. These evidences suggest that the neuronal activity of the PFC and hippocampus could be affected by iron homeostasis dysregulation in PAE rats.

### Iron Addition Decreased Basal Excitatory Transmission in the Scha-CA1 Region of the Hippocampus in Control But Not in PAE Rats

To evaluate whether altered iron homeostasis protein expression has any effects on synaptic transmission, we focused on glutamatergic neuronal activity in the hippocampal CA1 area, amply related with learning and memory processes (Eccles, 1986). We incubated hippocampal slices with Fe-NTA or DFP, in order to increase or deplete neuronal iron, respectively. Fe-NTA is a membrane impermeable iron complex, which can be uptake by cells in a transferrin-dependent or transferrin-independent manner (Matsuura, 1983; Chitambar and Sax, 1992). Meanwhile, DFP is a membrane permeable chelator for labile intracellular iron conforming the labile iron pool (LIP) (Glickstein et al., 2006).

First we evaluated basal excitatory synaptic transmission in hippocampal slices by measuring AMPAR field excitatory postsynaptic potentials (fEPSPs) in the CA1 region elicited by stimulation of Schaffer collateral fibers (Scha) before and after bath application of 20 µM Fe-NTA (**Figure 3A**). The two ANOVA anaysis revels a significant main effect between iron vs vehicle treatment [F_(1,5)_ = 20.72, p = 0.0061, η²p = 0.8] and an interaction between prenatal and iron treatment [F_(1,5)_ = 63.21, *p* = 0.0005, η²p = 0.98]. Analysis of the 10 last minutes of recording with Tukey’s *post-hoc* test reveled that Fe-NTA did not affect excitatory basal transmission in PAE animals (**Figure 3A**), meanwhile in control slices, Fe-NTA reduced significantly the AMPAR fEPSP compared with vehicle treatment (64.8 ± 4.5% C Fe vs 89.56 ± 2.1% C Veh) (p = 0.00046). We also analyzed the fiber volley (Fv) amplitude and PPR. The two way ANOVA analysis of Fv and PPR, reveled a significant main interaction between prenatal conditions with iron treatment for Fv: F_(1,5)_ = 13.013, p = 0.01542, η²p = 0.72, and for PPR: F_(1,5)_ = 20.10045, p = 0.0065, η²p = 0.8. Tukey’s *post-hoc* test was not able to show significantly differences, however, since the data are recolected before and after treatment, we performed a paired t test, which showed significant reduction in FV (0.014 ± 0.004, p = 0.00272 paired t test) together with a significant increased PPR (1.5 ± 0.029, p = 0.008 paired t test pre vs post application of Fe-NTA in control slices). These results showed that excitatory basal transmission was dysregulated in the hippocampal CA1 of PAE rats, since it was unaffected by Fe-NTA when transmission was reduced, most likely affecting presynaptic function.

Next we tested the efficacy of the glutamatergic synapse of PAE slices in presence of Fe-NTA. We measured AMPAR fEPSP at different stimulation intensities. No differences were detected in the fEPSP AMPAR responses (**Supplementary Figure 3**).

Additionally, we evaluated the effect of labile iron depletion on excitatory basal transmission in PAE and control rats with 100 µM DFP (**Figure 3B**). The two way ANOVA analysis showed a significant main differences between vehicle vs DFP treatment [F_(1,4)_ = 10.319, p = 0.003252, η²p = 0.72]. The Tukey analysis of the 10 last minutes of recording showed that DFP significant decreased AMPAR fEPSP in PAE rats (90.46 ± 2.27% PAE DFN vs 98.84 ± 2.03% PAE Veh (p = 0.03605), a pattern that was very similar, but not significant, to found in slices from control animals bathed with DFP (100.1 ± 2.16% C Veh; 92.18 ± 1.6% C DFN; p = 0.077). Because the trens obseved in the bar graph 3B we conducted a planned analysis between C Veh and C DFN. We found that DFN reduced AMPAR fEPSP in control slices (p = 0.015 unpaired t test). In addition, no significant differences were found in the Fv and PPR in PAE or controls before and after DFP application. We used vehicle (saline or DMSO) as a control of recording, demonstrating the stable recording during the whole experiment. These results suggest that a regulated LIP concentration is necessary to maintain AMPAR fEPSP in both conditions, PAE and control rats.

### Iron Addition Exacerbated the Decreasing Long-Term Potentiation in PAE Rats Compared to Control Rats

**Figure 4:**
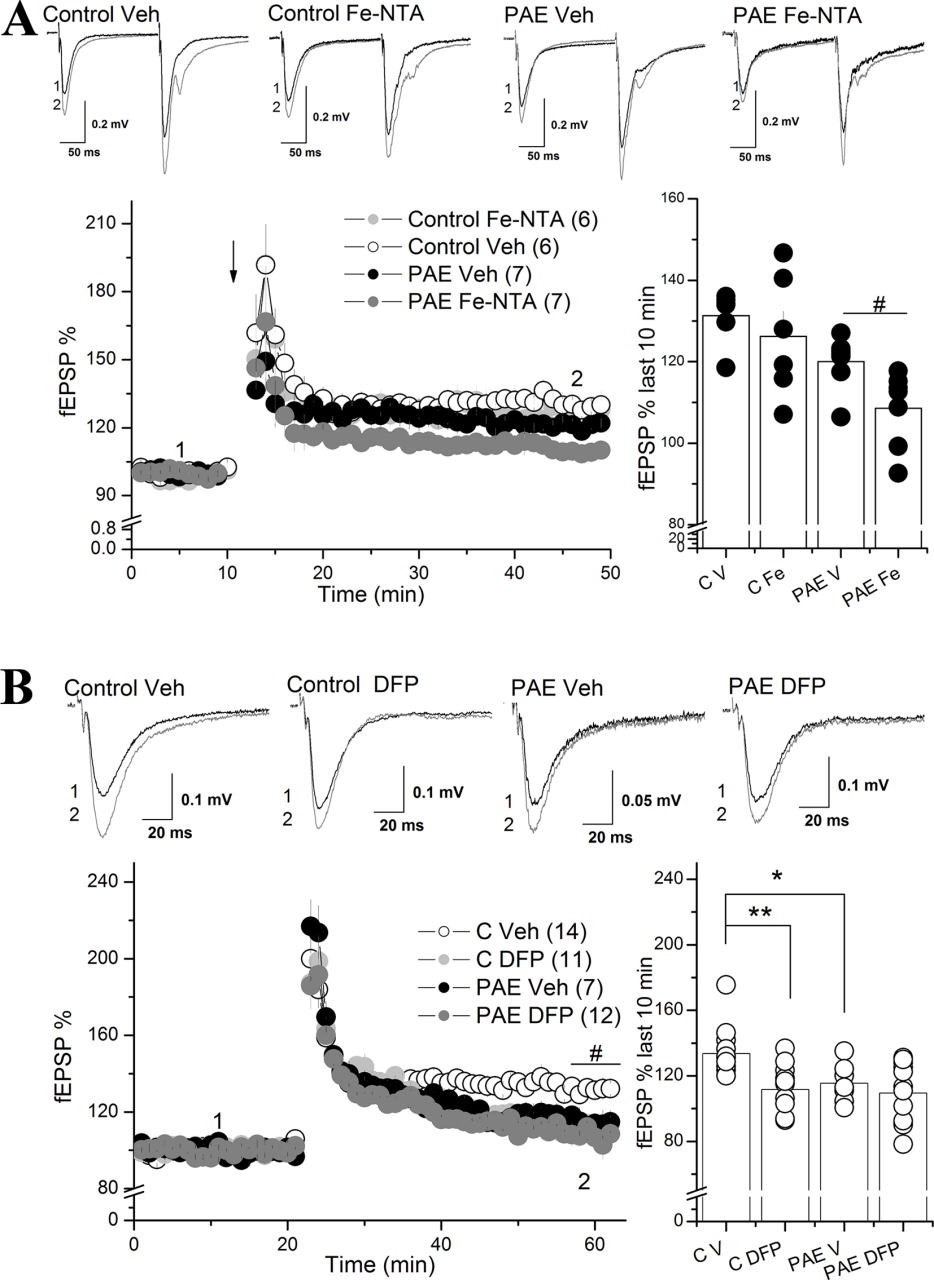
Iron addition (Fe-NTA) and iron chelation (DFP) altered the long-term potentiation (LTP) of prenatal ethanol exposure (PAE) rats compared to controls rats. **(A)** Effect of Fe-NTA on CA1 LTP of PAE and control rats. For long-term potentiation (LTP) analysis, slices from control or PAE rats were preincubated with 20 μM Fe-NTA, or saline (vehicle), during 40 min and continually perfused during the experiment. LTP (% fEPSP) was induced by high frequency stimulation (HFS) (100 Hz, 1 s). The average % fEPSP of the last 10 min was represented by bar graphs. Note that in PAE but not in control animals, Fe-NTA produced a significant decrease of LTP. **(B)** Effect of DFP on CA1 LTP of PAE and control rats. Slices were preincubated for 40 min with DFP and were continually perfused during the experiment. LTP experiment similar to A but in the presence of 100 μM of DFP. LTP (% fEPSP) was induced by high frequency stimulation (HFS) (100 Hz, 1 s). The average % fEPSP of the last 10 min was represented by bar graphs. Note that DFP significantly affected the LTP of control rats but not PAE rats. In all panels, sample traces were taken at the times indicated by numbers on the summary plot. The number of litter is indicated in parentheses. # Two way ANOVA C vs C DFP in temporal curse graph **(B)**.**p value < 0.01, *p value < 0.05, repeated measured two way ANOVA followed Tukey’s *post-hoc* test. ^#^p < 0.01 unpaired t test.

Previous studies have shown that intracellular iron participates in signaling mediated by ROS, which can modulate LTP in a dose-dependent manner (Massaad and Klann, 2011)(Beckhauser et al., 2016). Our results show that the hippocampus from PAE rats presented a misregulation in genes involved in iron homeostasis (TFR, FPN, and FT), predicting that LTP will be much more affected by incubation with iron than the control. To evaluate this we evoked LTP using HFS in the Sha-CA1 region from slices preincubated 40 min with 20 µM Fe-NTA or vehicle (saline) (**Figure 4A**).The two way ANOVA analysis showed a significant main differences between AMPAR fEPSP in slices from control vs PAE animals [F_(1,5)_ = 11.48 p = 0.01949, η²p = 0.69), as previously described (Marquardt and Brigman, 2016) (**Figure 4A**). Also is showed a significant difference between vehicle and Fe-NTA treatment [F_(1,5)_ = 4.767, p = 0.008076 η²p = 0.48). However, the Tukey analysis showed that iron incubation slighly decreased LTP in PAE rats compared to vehicle incubation (108.52 ± 3.48% PAE Fe; 119.99 ± 2.50% PAE Veh, Tukey p = 0.05706). We also conduced a planned comparision between both groups and found that Fe-NTA treatment significant reduced LTP in PAE animals (p = 0.0202 unpaired t test).

In contrast, Fe-NTA incubation did not affect LTP in control rats compared to control incubated only with vehicle ((126.23 ± 6.18% C Fe vs 131.29 ± 2.70% C veh, p = 0.4245 Tukey’s *post-hoc* test). These results showed that the imbalance in the iron proteins observed in the hippocampus of PAE rats can be perfectly correlated with altered synaptic plasticity observed after iron addition in PAE, which does not occur in slices from control rats incubated with iron.

To analyze the effect of iron on LTP induction we quantified the area under the curve during a HFS burst as a measurement of charge transfer. Not changes were detected in PAE slices treated with vehicle versus Fe-NTA (**Supplementary Figure 4**).

Labile iron depletion with DFP impairs LTP in hippocampal slices (Muñoz et al., 2011). The two way ANOVA analysis showed a significant main differences between vehicle vs DFP treatment [F_(1,6)_ = 18.16, p = 0.00581, η²p = 0.7516). Tukey’s *post-hoc* test confirm a significant difference between control and PAE slices treated with vehicle (p = 0.04) and also that DFP decreased the magnitude of LTP in control hippocampal slices compared to control slice treated with vehicle (111.74 ± 4.51 C Fe % vs 133.66 ± 3.79% C Veh, p = 0.00551), **Figure 4B**); meanwhile DFP maintained the magnitude of LTP in PAE rats compared to PAE incubated with vehicle (109.57 ± 5.28% PAE Fe NTA, 115.53 ± 4.51% PAE Veh, p = 0.15697). These results showed that the remained plasticity in PAE animals does not require intracellular labile iron levels in contrast to the iron requirements of control animals. Moreover, the addition of Fe in PAE slices also is detrimental for LTP, strongly suggesting altered iron homeostasis compared to controls.

Altogether, we found for the first time that rats with prenatal alcohol exposure presented misregulated expression of genes involved in iron homeostasis at the PFC (upregulation of DMT1) and the hippocampus (upregulation of TfR, and downregulation of FT and FPN), which could be functionally correlated with a dysregulated basal transmission and plasticity in the CA1 of PAE rats compared to controls.

## Discussion

PAE may generate fetal alcohol spectrum disorder (FASD) characterized by cognitive alterations in learning and memory formation (Sutherland et al., 1998; Patten et al., 2014; Contreras et al., 2017). Since the mechanisms are poorly understood, some recent studies have shown that PAE alters hippocampal synaptic plasticity (Marquardt and Brigman, 2016), and affects brain iron homeostasis (Miller et al., 1995; Carter et al., 2007; Rufer et al., 2012; Huebner et al., 2016), supporting the hypothesis that PAE can dysregulates brain iron homeostasis, contributing to altered hippocampal synaptic plasticity in adolescent animals. To evaluate this, we analyzed the expression of genes involved in iron homeostasis [DMT1, TFR, H-FT, FPN, and hepcidin (HAMP)] at three areas related with cognitive processes such as the PFC, hippocampus, and VTA (Lisman and Grace, 2005), together with functional measurement of glutamatergic synaptic transmission and plasticity in the hippocampal CA1 region. We found that PAE misregulated specifically and differentially the expression of genes involved in iron homeostasis in the PFC and hippocampus, but not in the VTA. Interestingly, in adolescent hippocampus, PAE upregulated TfR, but downregulated both FT and FPN, most likely resulting in a higher levels of intracellular iron. These results are consistent with an altered glutamatergic transmission and plasticity in response to iron or iron chelation, in hippocampal CA1 slices. These evidences show for the first time that PAE affects iron homeostasis and iron-dependent synaptic transmission and plasticity at the hippocampal CA1 area, suggesting that iron homeostasis dysregulation may be a critical target for developing therapeutic strategies for offspring suffering FASD.

### PAE Misregulated Genes Involved in Iron Homeostasis in the Hippocampus of Adolescent PAE Rats

Our first finding that PAE misregulated genes involved in iron homeostasis at the PFC and hippocampus, but not in the VTA, was supported by mRNA (RT-qPCR) and protein expression (Western-blotting) analysis. We found that PAE specifically upregulated the iron uptake proteins DMT1 at the PFC, and TfR at the hippocampus, while PAE downregulated both FPN mRNA at the PFC, and FT and FPN at the hippocampus, in all tested 21 day old offspring (**Figures 1** and **2**). Since, previous studies have shown that PAE present decreased iron levels at the cerebral cortex, but not at the subcortical forebrain and brainstem between P1 and P60–75 (Miller et al., 1995) our results open the question that PAE decreased the levels of LIP in specific brain areas, however still remain to be determinate. In agreement with our results, recently Huebner and coworkers showed that PAE rats, with an iron deficient diet, produced an exacerbated iron decrease and downregulation of FT in the whole brain at gestational day 20.5 (G20.5) (Huebner et al., 2016). However, contrary to our results, this group reported that PAE downregulated TfR protein and did not affect DMT1 and FPN protein expression, differences that could be explained by the age of the animal analyzed (G20.5) and/or the type of samples analyzed (total brain homogenate) (Huebner et al., 2016), meanwhile our analysis of expression levels (mRNA and protein) were performed in specific brain areas from postnatal 21-day-old rats.

Our observation of altered expression of genes involved in iron homeostasis, high TfR, and low FT and FPN, is consistent with a cellular response to low intracellular labile iron levels, which can be mediated by IRP. It is known that cells in order to uptake or release iron can coordinately modulate the expression of iron transporters (DMT1, TfR, FPN) and iron-storage proteins (FT) at the transcriptional and translational levels (Hentze and Kuhn, 1996; Kruer, 2013). In low iron level conditions, cells increase their expression of TFR and DMT1 through IRP interacting with the 3′ mRNA of TfR and DMT1 to increase their stability and translation, while decreasing the expression of FPN and FT by binding IRP with IREs located at the 5′ of these mRNAs to repress translation (Bogdan et al., 2016). These antecedents suggest that the iron/IRP mechanism could be involved in the misregulation that we observed in some genes involved in iron homeostasis at the PFC and/or hippocampus of adolescent PAE rats. However, upregulation of DMT1-1B, an isoform lacking the IRE element (**Figures 1A, C**), suggests that DMT1 1B isoforms expression could be activated by a mechanism independent of IRP. A possible mechanism could be mediated by HAMP, which downregulates DMT-1B (−) IRE mRNA in the PFC (Li et al., 2011), predicting that low expression of hepcidin in the PAE could upregulates DMT-1B (−) IRE mRNA. However, contrary to this prediction, previous reports have shown that PAE rats (at gestational day 20) overexpress HAMP (Huebner et al., 2016), and also our RT-qPCR results show that HAMP mRNA expression was unaffected in all brain areas analyzed (**Figure 2**). The mechanisms involved in DMT1 overexpression in PAE animals is still unknown. However it is tempted to suggest a regulation by inflammatory molecules (IL6 and TNF) as it has been described to upregulate DMT1 in brain (Urrutia et al., 2013). More studies are necessary to resolve how PAE upregulates DMT1/(−)IRE. Thus, our results show that PAE specifically and differentially misregulated the expression of genes involved in iron homeostasis (TfR, DMT1B/(–)IRE, FT, and FPN) in brain areas related with learning and memory such as the PFC and hippocampus, but not in the VTA, suggesting iron homeostasis dysregulation as a new mechanism of maladapted neuronal plasticity in animals exposed to ethanol in utero. In addition, misregulation of genes involved in iron homeostasis observed in PAE animals could affect several mechanisms involved in synaptic transmission and plasticity that underlie cognitive alterations observed in FASD.

### PAE Presented Dysregulated Basal Hippocampal Transmission and Synaptic Plasticity That Correlates With Misregulation of Genes Involved in Iron Homeostasis

Several mechanisms may be involved in how iron homeostasis dysregulation in PAE rats that could affect the offspring and induce a maladaptation to the environment. Nutritional studies have shown that iron deficiency both prenatally and perinatally are associated with intellectual disabilities and psychiatric disorders (Carlson et al., 2007; Sidrak et al., 2014). Here we observed iron homeostasis genes are differentially regulated by PAE in several regions of the brain, perhaps modulating a coordinated activation of different regions involved in cognition, in special between PFC and hippocampus, where we found the more consistent observations described above. Moreover an iron deficient diet can affect hippocampal plasticity by impairing dopaminergic-mediated synaptic plasticity (Breton et al., 2015), disruption of synaptic maturation (Jorgenson et al., 2005), or by abnormality of the CA1 apical dendrite structure (Fretham et al., 2011; Muñoz and Humeres, 2012).

Also, the hippocampal conditional knockout mice (KO) of DMT1 (Slc11a2^hipp/hipp^) had a decreased energy status that promotes shorter dendrites and disorganized branching patterns in the hippocampal CA1 region compared with normal mice (Carlson et al., 2009), and also a reduced spatial memory behavior (Carlson et al., 2010). Also, iron homeostasis seems to be involved in hippocampal glutamatergic transmission, since DMT1B (mRNA and protein levels) was upregulated after NMDAR activation or spatial memory training (Haeger et al., 2010). Interestingly, recent evidence showed that iron uptake mediated by lysosomal DMT1 can negatively modulate NMDAR activity (White et al., 2016). Additionally, Liu and coworkers using a conditional KO mice for TfR, showed a dramatic reduction of basal transmission and long term plasticity (LTP) through an iron-independent mechanism involving TfR-dependent AMPAR trafficking (Liu et al., 2016). Therefore, all these studies, together with our gene expression results, suggest that this misregulation of the iron homeostasis genes could mediate altered glutamatergic transmission in the hippocampal CA1 region of PAE rats.

In agreement with this, our first electrophysiological results showed that AMPAR synaptic transmission in the hippocampal CA1 area of control rats was reduced together with decreased fiber volley and increased PPR when incubated with iron (Fe-NTA), suggesting an Iron dependent presynaptic mechanism. This evidence suggests that in controls, but not in PAE animals, Iron regulates glutamatergic transmission through a presynatic mechanism that in turn decreases neuronal excitability when exposed to excess iron. Nevertheless, this 40% of reduction in AMPAR synaptic transmission is not enough to affect the induction of long term potentiation in control animals in presence of Fe-NTA (**Figure 3A**).

Even though the mechanism affecting iron over the short term is unknown it has been described that synaptosome treated with 10 µM FeSO4 for 4 h decreases glutamate uptake (Guo et al., 2000). We can speculate that the elevated postsynaptic expression of TfR in PAE animals masks the presynaptic effect because it regulates the iron concentration in the synaptic cleft. However, deferiprone incubation slightly decreased AMPAR synaptic transmission in control as well in PAE animals denoting that the mechanism involving basal synaptic transmission is a complex process, therefore we cannot discard that other mechanisms are affecting by Iron, such as presynaptic and postsynaptic events (Vargas et al., 2007; Fioravante and Regehr, 2011; Ramaswami et al., 2016) and the complex regulation by astrocytes (Navarrete and Araque, 2011; Panatier et al., 2011). Additional studies are necessary to determine which is the putative mechanism.

Our second electrophysiological results showed that hippocampal synaptic plasticity in the CA1 was also altered by iron incubation (Fe-NTA) in adolescent PAE rats compared with controls (**Figures 4A, B**). Our findings showed that Fe-NTA exacerbated the impaired LTP in PAE rats compared to control animals. Since it is known that Fe-NTA can be uptake by the cell by Tf/TfR endocytic pathway and the Tf-independent mechanism (Matsuura, 1983)(Chitambar and Sax, 1992), it is tempting to suggest that the high expression of TfR and low expression of FT and FPN in the hippocampus of PAE animals allows intracellular iron levels to increase in turn impairing LTP. The changes in LIP could affect synaptic plasticity at the CA1 of PAE rats by different nonexclusive mechanisms.

One possibility is that changes in labile iron concentration affect NMDAR activity. In agreement with this, it was recently described that depletion of iron with a permeable chelator (pyridoxal isonicotinoyl hydrazine) enhances the excitability of hippocampal pyramidal cells, increasing the amount of the NR2A subunit and NMDAR activity (White et al., 2016). Interestingly, this iron-dependent NMDAR modulation requires iron release from lysosomes, which is a DMT1 mediated process (White et al., 2016). Given that an important pathway for iron uptake and release to the endosomal/lysosomal system is mediated by endocytosis of the Tf/TfR complex and lysosomal DMT1 (Fleming et al., 1998; Lawrence et al., 1999; Cheng et al., 2004), the upregulated TfR expression in the hippocampus of PAE rats could promote increased iron uptake, after incubation with Fe-NTA, compared to controls; producing the lower LTP observed in the hippocampal CA1 of PAE rats.

An additional mechanism that could be involved in impaired LTP at the CA1 of PAE rats after incubation with Fe-NTA may be mediated by oxidative stress. A controlled ROS production provides the optimal redox state necessary for synaptic plasticity (Beckhauser et al., 2016), however when high ROS is accumulated it induces oxidative stress which is associated with LTP impairment and cognitive decline as observed in neurodegenerative disorders and age-dependent decay of neuroplasticity (Massaad and Klann, 2011). Physiological intracellular iron levels can react with peroxide (H_2_O_2_), through the Haber–Weiss and Fenton reactions, to produce hydroxyl radical which sensitizes ryanodine receptor, a endoplasmatic Ca^2+^ channels, to calcium induces calcium release after activation of NMDAR during induction of LTP (Muñoz et al., 2011). Additionally, the incubation of several iron-related compounds such as forms of hemoglobin, hemin, and ferrous chloride increase ROS, affecting synaptic transmission of hippocampal brain slices (Pellmar, 1986; Pellmar et al., 1991; Yip and Sastry, 2000). Thus, these evidences suggest that incubation with Fe-NTA promotes high intracellular iron levels, mediated by dysregulated expression of genes involved in iron homeostasis (TFR, FPN, and FT), favoring the Fenton reaction and oxidative stress, impairing LTP. Several oxidative stress mechanisms could affect hippocampal LTP, including ROS mediated upregulation of calcineurin, which through the activation of protein phosphatase 1 can decrease AMPAR activity, and/or ROS mediated modulation of CAMKII that can directly alters NMDA receptor function (Beckhauser et al., 2016).

It is amply known that iron-dependent ROS generation regulates NMDAR activity, favoring NMDAR-induced calcium signal (Muñoz et al., 2011) or negatively modulate NMDAR activity mediated by lysosomal iron uptake througth DMT1 (White et al., 2016). In our case, exogenous iron application, but not iron chelation, decreased LTP in PAE slices. It is tempting to suggest that mysregulated iron homeostasis in PAE animals could affect iron-dependent NMDAR activation during LTP induction. However, quantification of the area under HFS-burst does not change in the the presence of Iron (**Supplementary Figure 4**), supporting the idea that the global machinery during LTP induction is not modified. However, we can still not discard a direct and a transient effect on NMDAR activity.More studies are necessary to dissect the particular role of iron in the hippocampal plasticity of PAE animals.

Similar to the hippocampus, we detected changes in the iron transporter DMT1 and FNP in PFC at P21, but not in P70–78 or VTA. However, we did not evaluate the functional significance of the mysregulated iron homeostasis in these brain region. We speculate that PFC function could be similar to the hippocampus due to their active metabolic rate (Rice and Barone, 2000) and complementarity in memory processes (Nelson, 1995). Moreover, perinatal iron deficiency increased the expression of iron transporters in both areas, similar to that observed in this study. In contrast striatum did not have the same response as the hippocampus and cortex, suggesting different iron requirements for striatum activity (Siddappa et al., 2003). Moreover the function of the PFC and hippocampus was measured by different tasks in adolescents with chronic and severe iron deficiency during infancy. They showed impairment in the frontostriatal-mediated executive functions and in the performance of hippocampus-based recognition memory tasks (Lukowski et al., 2010) supporting similar Iron requirement in both areas.

In the VTA of PAE animals iron homeostasis genes did not change compared to age-matched controls (P21 and P70–78). However we did observe that the VTA of control animals expressed higher levels of DMT1(+), IRE, DMT1 (−) IRE y HAMP than the hippocampus, and higher levels of HAMP mRNA than the PFC, respectively (see **Supplementary Figure 1**, **Supplementary Table 1A**). These evidences complement the essential role of iron for the synthesis, metabolism, and function of dopamine (Ramsey et al., 1996; Matak et al., 2016; Yien and Paw, 2016). Regarding the function of VTA in PAE animals, we previously described that NOX2 inhibition, a synaptic enzyme that synthesizes superoxide in the VTA, restored alcohol-conditioned place preference in PAE animals (Contreras et al., 2017). Moreover we detected higher DOPAC levels, a dopamine metabolite, in the VTA terminals of nucleus accumbens of PAE animals (Plaza et al., 2019). However, the mechanism involved in iron homeostasis and the particular role of Iron in VTA function of PAE animals will be a matter to be addressed in future studies.

The present study has several limitations and caveats. Since changes in the expression of different mRNAs involved in iron homeostasis suggest reduced intracellular iron levels in the hippocampus of PAE animals, we also need to measure iron as well protein levels in different brain regions. Moreover, to understand the iron-dependent mechanism involved in glutamatergic transmission it will be important to study the role of Iron in isolated NMDAR activity and its participation in hippocampal LTP induction.

Despite these limitations, the present study showed, for the first time, that adolescents rat that were exposed to alcohol *in utero* presented a specific misregulation of iron homeostasis genes (DMT1, TFR, FT, and FPN) in the PFC and hippocampus, but not in the VTA. Moreover, in the hippocampal CA1 area of PAE rats we found a functional correlation between a misregulation of iron homeostasis genes with dysregulated synaptic transmission and plasticity of this brain area. Alterations in iron homeostasis could represent a potential mechanism that underlies the cognitive deficits associated with FASD. In addition, our study predicts that nutritional iron supplementation of postnatal PAE animals could be deleterious for neuronal activity and plasticity, since it could add to the potential increase of iron mediated by the misregulation of iron homeostasis genes. Identification of this mechanism will facilitate the development of novel therapies for fetal alcohol spectrum disorder.

## Data Availability Statement

All datasets generated for this study are included in the article/**Supplementary Material**.

## Ethics Statement

The animal study was reviewed and approved by Bioethic, Scientific, and Animal Care and Use Committee of the Universidad Católica del Norte, Chile.

## Author Contributions

EF-O and PH contributed to the conception and design of the study. EF-O and SV-R contributed to the acquisition and statistical analysis of RT-qPCR experiments. WP-B and PH contributed to the acquisition of data and statistical analysis electrophysiological experiments. EF-O and PH worked on the analysis and interpretation of data and wrote the manuscript. All authors contributed to manuscript revision, read, and approved the submitted version.

## Funding

This work was supported by Chilean Fondecyt grant no. 1140855 and from PEW Latin American postdoctoral fellowship to P.H. VRIDT-UCN, IBRO-LARC.

## Conflict of Interest

The authors declare that the research was conducted in the absence of any commercial or financial relationships that could be construed as a potential conflict of interest.

## Abbreviations

VTA, ventral tegmental area; PFC, prefrontal cortex; TfR, transferrin receptor; DMT1, divalent metal transporter; FT, ferritin; FPN, ferroportin; P21, postnatal day 21; P70–78, postnatal day 70–78.
