# Supplementary figures and images for: Prenatal Ethanol Exposure Misregulates Genes Involved in Iron Homeostasis Promoting a Maladaptation of Iron Dependent Hippocampal Synaptic Transmission and Plasticity
Source: Front Pharmacol. 2019 Nov 7;10:1312. doi: 10.3389/fphar.2019.01312 (PMC6855190; doi:10.3389/fphar.2019.01312)

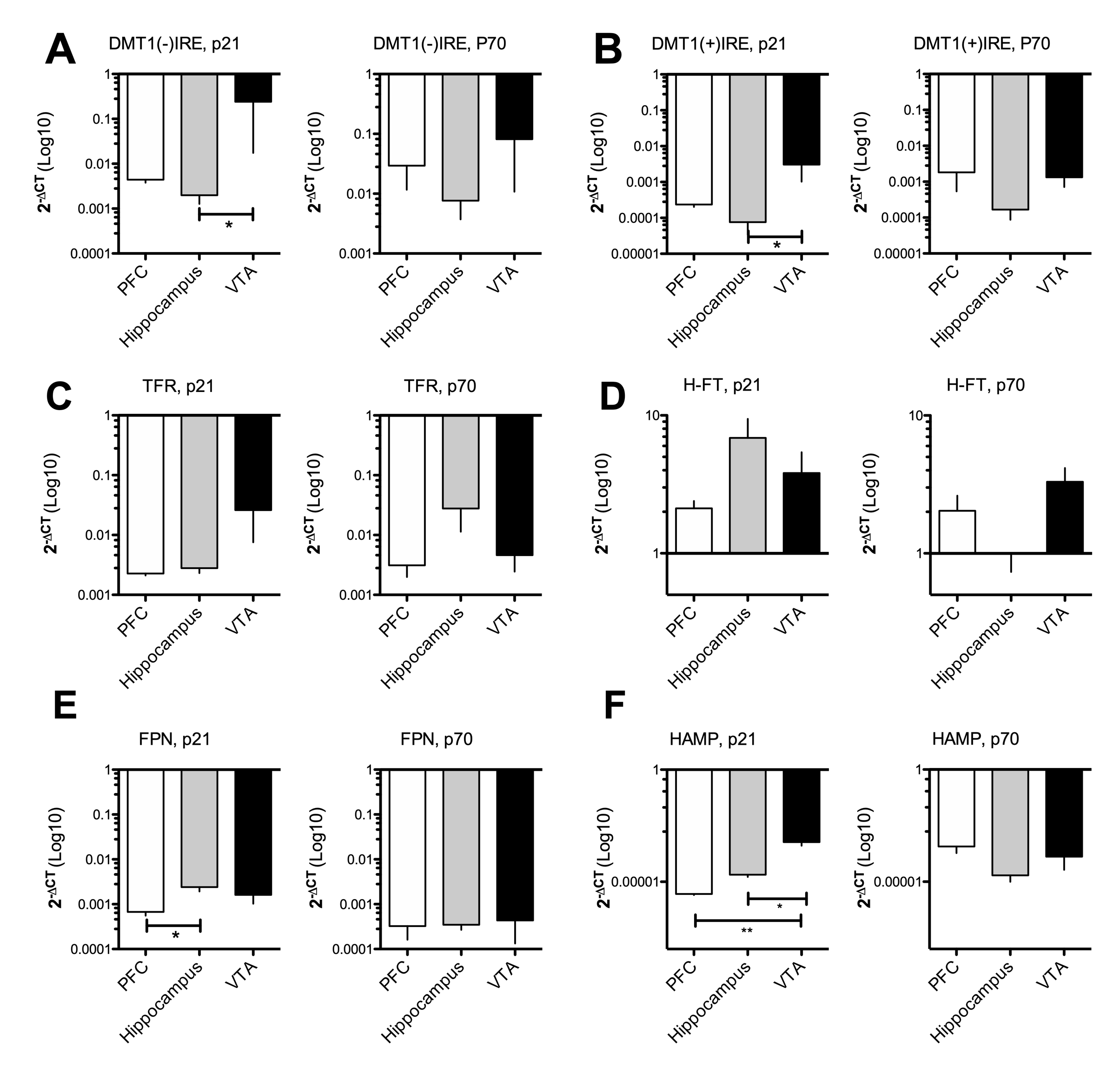

Supplement: Supplementary file 1 [file Image_1.tiff]

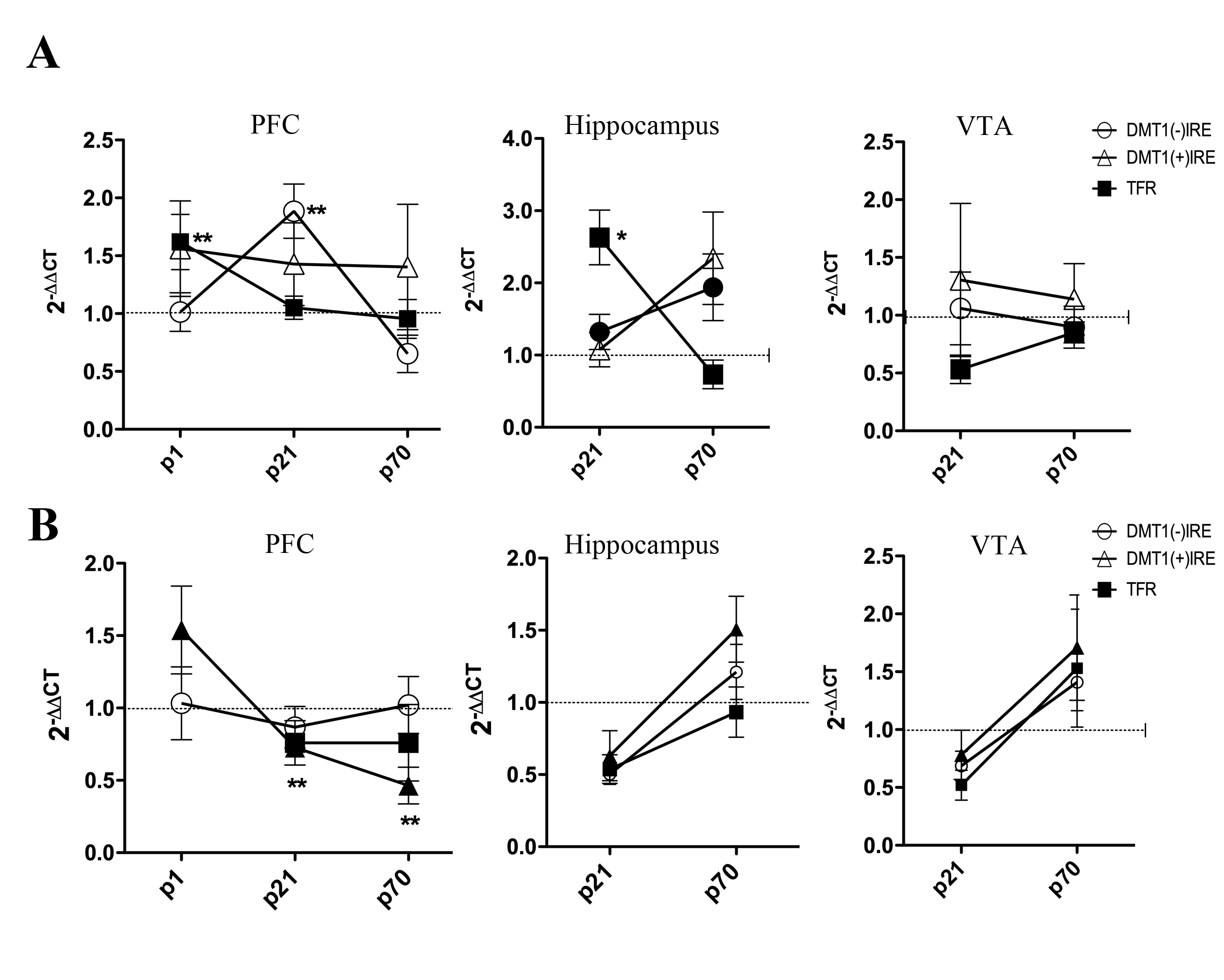

Supplement: Supplementary file 2 [file Image_2.tif]
