# Supplementary material for: Prenatal Ethanol Exposure Misregulates Genes Involved in Iron Homeostasis Promoting a Maladaptation of Iron Dependent Hippocampal Synaptic Transmission and Plasticity
Source: Front Pharmacol. 2019 Nov 7;10:1312. doi: 10.3389/fphar.2019.01312 (PMC6855190; doi:10.3389/fphar.2019.01312)
Supplement: Supplementary file 5 [file Table_1.docx]

***Supplementary Tables 1***

***1A.*** *Comparison of mRNA expression of iron homeostasis genes in control rats between different brain areas (PFC, Hippocampus and VTA) at P21 or P70-78.* Analysis with nonparametric one way ANOVA (Kruskal-Wallis) followed by Dunn's Multiple Comparison Test.

| **Iron Homeostasis Gene** | **Kruskal-Wallis statistic,PFC, Hippocampus and VTA** | ***p value***  **Comparison: PFC, Hippocampus and VTA** | **Dunn's Multiple Comparison Test, "Significant? **p* < 0.05,  *** p* < 0.01, **** p* < 0.001?"** |
| --- | --- | --- | --- |
| DMT1(-) IRE, p21 | 9.225 | 0.0099 | Hippocampus-21 vs VTA-21, yes **. |
| DMT1(-) IRE, p70 | 1.893 | 0.3881 | NS |
| DMT1(+) IRE, p21 | 15.17 | 0.0005 | Hippocampus-21 vs VTA-21, yes ***. |
| DMT1(+) IRE, p70 | 4.774 | 0.0919 | NS |
| TFR, p21 | 3.993 | 0.1358 | NS |
| TFR, p70 | 0.5812 | 0.7478 | NS |
| H-FT, p21 | 5.384 | 0.0678 | NS |
| H-FT, p70 | 5.568 | 0.0618 | NS |
| FPN, p21 | 7.505 | 0.0235 | NS |
| FPN, p70 | 1.399 | 0.4968 | NS |
| HAMP, p21 | 19.48 | < 0.0001 | PFC-21 vs VTA-21, yes ***. |
|  |  |  | Hippocampus vs VTA, yes. |
| HAMP, p70 | 3.171 | 0.2049 | NS |

***Table 1B,*** *Comparison of mRNA expression* (**2^-ΔCt^)** *of iron homeostasis genes in control rats between differents ages P21 and P70-78* at specific brain areas (PFC, Hippocampus and VTA). Analysis Mann Whitney test.

| **Iron homeostasis gene** | **Brain Area** | **P21, Mean ± SEM**  **2^-ΔCT^** | **P70-P78, Mean ± SEM**  **2^-ΔCT^** | ***p value*** | **Mann-Whitney U** | **Signif. Different (*p < 0.05)** |
| --- | --- | --- | --- | --- | --- | --- |
| DMT1 (-) IRE | PFC | 0.005576 ± 0.001422 N=3 | 0.02587 ± 0.02361 N=4 | 0.7857 | 6 | no |
|  | Hippocampus | 0.002760 ± 0.001604 N=4 | 0.004880 ± 0.002823 N=5 | 0.9048 | 9 | no |
|  | VTA | 0.6186 ± 0.5919 N=3 | 0.09409 ± 0.08540 N=5 | 0.3929 | 4 | no |
| DMT1 (+) IRE | PFC | 0.0002856 ± 6.101e-005 N=3 | 0.001302 ± 0.001108 N=5 | 0.7857 | 6 | no |
|  | Hippocampus | 8.085e-005 ± 6.080e-005 N=4 | 0.0001727 ± 8.936e-005 N=5 | 0.5556 | 7 | no |
|  | VTA | 0.005051 ± 0.003158 N=3 | 0.0008571 ± 0.0003731 N=5 | 0.1429 | 2 | no |
| TFR | PFC | 0.002491 ± 0.0002819 N=3 | 0.002471 ± 0.0005857 N=5 | 0.7857 | 6 | no |
|  | Hippocampus | 0.003330 ± 0.0009670 N=4 | 0.01883 ± 0.01513 N=5 | 0.5556 | 7 | no |
|  | VTA | 0.05887 ± 0.04740 N=3 | 0.004568 ± 0.002705 N=5 | 0.0714 | 1 | no |
| H-FT | PFC | 2.363 ± 0.3153 N=3 | 1.556 ± 0.3736 N=5 | 0.1429 | 2 | no |
|  | Hippocampus | 2.858 ± 1.201 N=4 | 0.9098 ± 0.2455 N=5 | 0.2857 | 5 | no |
|  | VTA | 2.678 ± 0.6801 N=3 | 2.446 ± 0.9519 N=5 | 1 | 7 | no |
| FPN | PFC | 0.0008374 ± 0.0001928 N=3 | 0.0002608 ± 0.0001707 N=5 | 0.1429 | 2 | no |
|  | Hippocampus | 0.002581 ± 0.0005899 N=4 | 0.0002985 ± 8.635e-005 N=5 | 0.0159 | 0 | yes * |
|  | VTA | 0.0009237 ± 0.0003728 N=3 | 0.0005326 ± 0.0003503 N=5 | 0.3929 | 4 | no |
| HAMP | PFC | 2.926e-006 ± 1.448e-007 N=3 | 0.0002551 ± 0.0001733 N=5 | 0.0357 | 0 | yes * |
|  | Hippocampus | 2.470e-005 ± 6.641e-006 N=4 | 2.340e-005 ± 6.385e-006 N=5 | 0.9048 | 9 | no |
|  | VTA | 0.0006212 ± 0.0002670 N=3 | 0.0001560 ± 0.0001162 N=5 | 0.0714 | 1 | no |
