# Supplementary material for: Prenatal Ethanol Exposure Misregulates Genes Involved in Iron Homeostasis Promoting a Maladaptation of Iron Dependent Hippocampal Synaptic Transmission and Plasticity
Source: Front Pharmacol. 2019 Nov 7;10:1312. doi: 10.3389/fphar.2019.01312 (PMC6855190; doi:10.3389/fphar.2019.01312)
Supplement: Supplementary file 6 [file Table_2.docx]

***Supplementary Tables 2:***

A) Comparison of mRNA expression (**2^-ΔCt control or PAE^**/**2^-ΔCt mean control^ )** of Iron homeostasis genes (DMT1 (-) IRE, DMT1 (+) IRE and TFR) between PAE and control rats at P21 age. Analysis Mann Whitney test.

| **Brain Area** | **Iron Homeostasis Genes** | **Control Rats Mean ± SEM, N** | **PAE rats Mean ± SEM, N** | ***p value*** | **Mann-Whitney U** | **Signif. Different (**p < 0.05,* ***p < 0.05*)** |
| --- | --- | --- | --- | --- | --- | --- |
| **PFC** | DMT1 (-) IRE | 1.001 ± 0.1438 N=8 | 1.925 ± 0.3825 N=8 | 0.0415 | 15 | yes* |
|  | DMT1 (+) IRE | 1.000 ± 0.1464 N=8 | 1.375 ± 0.3726 N=8 | 0.3227 | 27 | no |
|  | TFR | 1.001 ± 0.06960 N=8 | 1.063 ± 0.1179 N=8 | 0.4373 | 30 | no |
| **Hippocampus** | DMT1 (-) IRE | 1.000 ± 0.3621 N=9 | 0.8511 ± 0.2684 N=9 | 0.3619 | 36 | no |
|  | DMT1 (+) IRE | 1.000 ± 0.6328 N=5 | 1.628 ± 1.048 N=5 | 0.3766 | 10,5 | no |
|  | TFR | 1.000 ± 0.1757 N=9 | 2.818 ± 0.7804 N=9 | 0.0071 | 13 | yes ** |
| **VTA** | DMT1 (-) IRE | 0.9992 ± 0.5041 N=5 | 0.6720 ± 0.3424 N=5 | 0.4206 | 11 | no |
|  | DMT1 (+) IRE | 0.9994 ± 0.6655 N=5 | 1.052 ± 0.5447 N=5 | 0.4206 | 11 | no |
|  | TFR | 0.9994 ± 0.7090 N=8 | 1.711 ± 0.7497 N=8 | 0.2209 | 24 | no |

**2B)** Comparison of mRNA expression (**2^-ΔCt control or PAE^**/**2^-ΔCt mean control^ )** of Iron homeostasis genes (DMT1 (-) IRE, DMT1 (+) IRE and R-TFR) between PAE and control rats at P70-78 age. Mann Whitney test.

| **Brain Area** | **Iron Homeostasis Genes** | **Control Rats Mean ± SEM, N** | **PAE Rats Mean ± SEM, N** | ***p value*** | **Mann-Whitney U** | **Signif. Different (**p < 0.05,* ***p < 0.05*)** |
| --- | --- | --- | --- | --- | --- | --- |
| **PFC** | DMT1 (-) IRE | 1.000 ± 0.6049 N=7 | 0.6469 ± 0.3810 N=7 | 0.1914 | 17 | no |
|  | DMT1 (+) IRE | 1.000 ± 0.7031 N=7 | 2.334 ± 2.158 N=7 | 0.4508 | 23 | no |
|  | TFR | 1.000 ± 0.3621 N=7 | 0.7804 ± 0.2697 N=7 | 0.4508 | 23 | no |
| **Hippocampus** | DMT1 (-) IRE | 1.000 ± 0.5133 N=6 | 0.8935 ± 0.5022 N=6 | 0.4091 | 16 | no |
|  | DMT1 (+) IRE | 1.000 ± 0.4811 N=6 | 1.263 ± 0.4406 N=6 | 0.2424 | 13 | no |
|  | TFR | 1.000 ± 0.5903 N=6 | 0.8459 ± 0.6840 N=6 | 0.4091 | 16 | no |
| **VTA** | DMT1 (-) IRE | 1.000 ± 0.8679 N=6 | 0.8574 ± 0.7629 N=6 | 0.4091 | 16 | no |
|  | DMT1 (+) IRE | 1.000 ± 0.4674 N=6 | 0.8750 ± 0.1932 N=6 | 0.2944 | 14 | no |
|  | TFR | 1.000 ± 0.4703 N=6 | 0.8857 ± 0.4820 N=6 | 0.2424 | 13 | no |
