# Supplementary material for: Prenatal Ethanol Exposure Misregulates Genes Involved in Iron Homeostasis Promoting a Maladaptation of Iron Dependent Hippocampal Synaptic Transmission and Plasticity
Source: Front Pharmacol. 2019 Nov 7;10:1312. doi: 10.3389/fphar.2019.01312 (PMC6855190; doi:10.3389/fphar.2019.01312)
Supplement: Supplementary file 7 [file Table_3.docx]

***Supplementary Tables 3:***

***A)*** Comparison of mRNA expression (**2^-ΔCt control or PAE^**/**2^-ΔCt mean control^ )** of Iron homeostasis genes (H-FT, FPN and HAMP) between PAE and control rats at P21 age. Mann Whitney test.

| **Brain Area** | **Iron Homeostasis Genes** | **Control Rats**  **Mean ± SEM, N** | **PAE Rats Mean ± SEM, N** | ***p value*** | **Mann-Whitney U** | **Signif. Different (P < 0.05)** |
| --- | --- | --- | --- | --- | --- | --- |
| **PFC** | H-FT | 0.9980 ± 0.1308 N=8 | 0.8673 ± 0.2055 N=8 | 0.300 | 22 | no |
|  | FPN | 0.9963 ± 0.1759 N=8 | 0.6738 ± 0.08506 N=8 | 0.0415 | 15 | yes |
|  | HAMP | 0.9801 ± 0.09637 N=8 | 0.7338 ± 0.1546 N=8 | 0.2209 | 24 | no |
| **Hippocampus** | H-FT | 0.9997 ± 0.3728 N=9 | 0.4513 ± 0.1710 N=9 | 0.0122 | 15 | yes |
|  | FPN | 0.9996 ± 0.2025 N=8 | 0.5541 ± 0.2172 N=8 | 0.019 | 12 | yes |
|  | HAMP | 0.9996 ± 0.2095 N=11 | 0.7336 ± 0.3121 N=9 | 0.0983 | 32 | no |
| **VTA** | H-FT | 0.9991 ± 0.4155 N=8 | 0.5274 ± 0.1547 N=8 | 0.2869 | 26 | no |
|  | FPN | 0.9995 ± 0.3641 N=8 | 1.435 ± 0.7574 N=8 | 0.4392 | 30 | no |
|  | HAMP | 0.9995 ± 0.3060 N=8 | 0.6553 ± 0.2310 N=8 | 0.2869 | 26 | no |

**B)** Comparison of mRNA expression (**2^-ΔCt control or PAE^**/**2^-ΔCt mean control^ )** of Iron homeostasis genes (H-FT, FPN and HAMP) between PAE and control rats at P70-78 age. Mann Whitney test.

| **Brain Area** | **Iron Homeostasis Genes** | **Control Rats**  **Mean ± SEM, N** | **PAE Rats**  **Mean ± SEM, N** | ***p valu*e** | **Mann-Whitney U** | **Signif. Different (P < 0.05)** |
| --- | --- | --- | --- | --- | --- | --- |
| **PFC** | H-FT | 1.000 ± 0.2303 N=4 | 0.8125 ± 0.04442 N=4 | 0.1714 | 4 | no |
|  | FPN | 1.000 ± 0.6840 N=4 | 1.564 ± 1.384 N=4 | 0.3429 | 6 | no |
|  | HAMP | 1.000 ± 0.4912 N=7 | 0.8934 ± 0.7774 N=7 | 0.1914 | 17 | no |
| **Hippocampus** | H-FT | 1.000 ± 0.2602 N=6 | 1.163 ± 0.3356 N=6 | 0.4686 | 17 | no |
|  | FPN | 1.000 ± 0.2309 N=6 | 1.052 ± 0.2717 N=6 | 0,5 | 18 | no |
|  | HAMP | 1.000 ± 0.4849 N=6 | 1.264 ± 0.4796 N=6 | 0.3496 | 15 | no |
| **VTA** | H-FT | 1.000 ± 0.2608 N=6 | 1.417 ± 0.5522 N=6 | 0.4091 | 16 | no |
|  | FPN | 1.000 ± 0.6927 N=6 | 0.5471 ± 0.2930 N=6 | 0.4091 | 16 | no |
|  | HAMP | 1.000 ± 0.7447 N=6 | 1.129 ± 0.8467 N=6 | 0.2944 | 14 | no |
