# Supplementary material for: Prenatal Ethanol Exposure Misregulates Genes Involved in Iron Homeostasis Promoting a Maladaptation of Iron Dependent Hippocampal Synaptic Transmission and Plasticity
Source: Front Pharmacol. 2019 Nov 7;10:1312. doi: 10.3389/fphar.2019.01312 (PMC6855190; doi:10.3389/fphar.2019.01312)
Supplement: Supplementary file 8 [file DataSheet_1.pdf]

# WESTERN BLOT ANTI-DMT1 IN HIPPOCAMPUS

Control Samples (C), PAE samples (Ethanol, E)

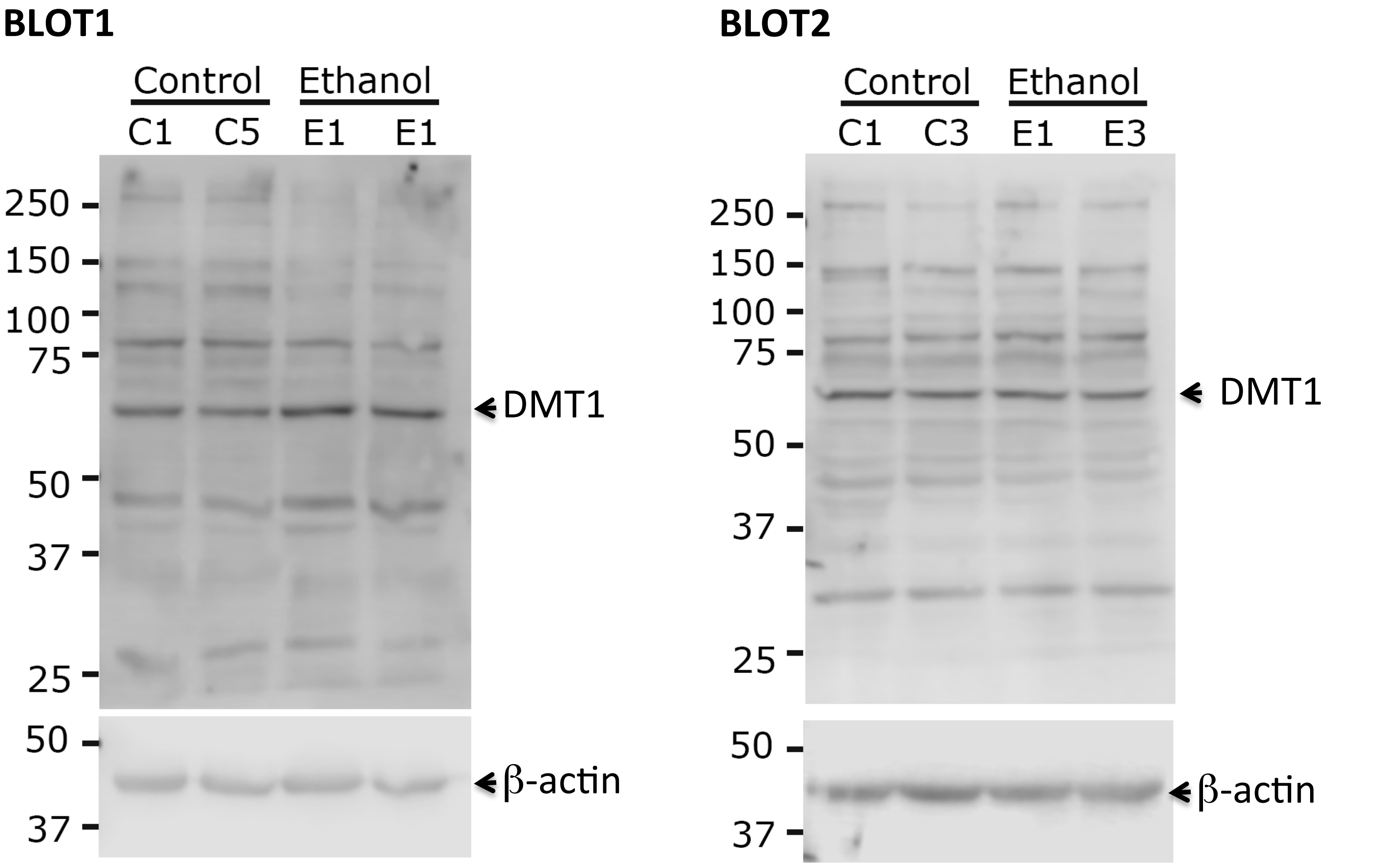


Hippocampus, control samples n=4, PAE samples n=4

## WESTERN BLOT ANTI-DMT1 IN PREFROLTAL CORTEX (PFC) AND VENTRAL TEGMENTAL AREA (VTA)

Control Samples (C), PAE samples (E)

**BLOT3**

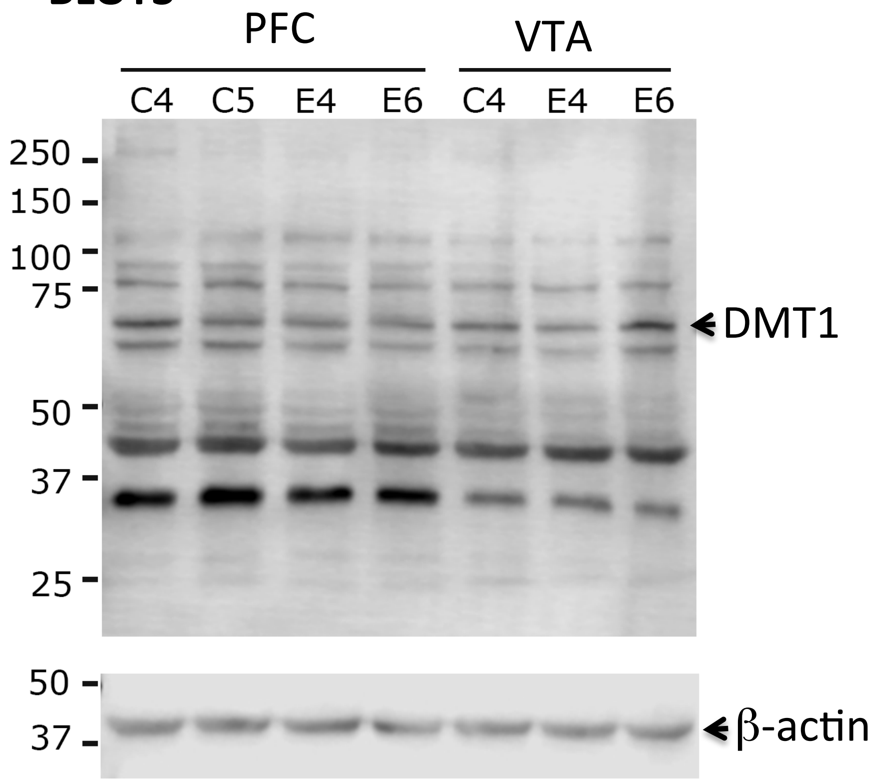


**BLOT4**

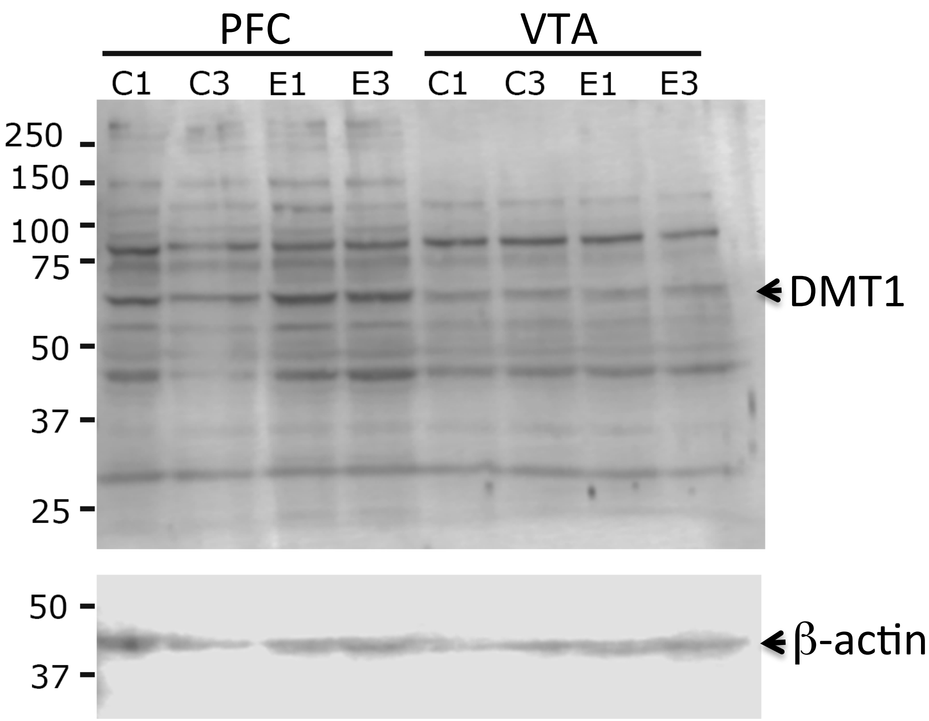


PFC, control samples n=4, PAE samples n=4
VTA, control samples n=3, PAE samples n=4

# WESTERN BLOT ANTI-DMT1 IN PREFROLTAL CORTEX (PFC) AND (VENTRAL TEGMENTAL AREA) VTA

Control Samples (C), PAE samples (E)

**BLOT5**

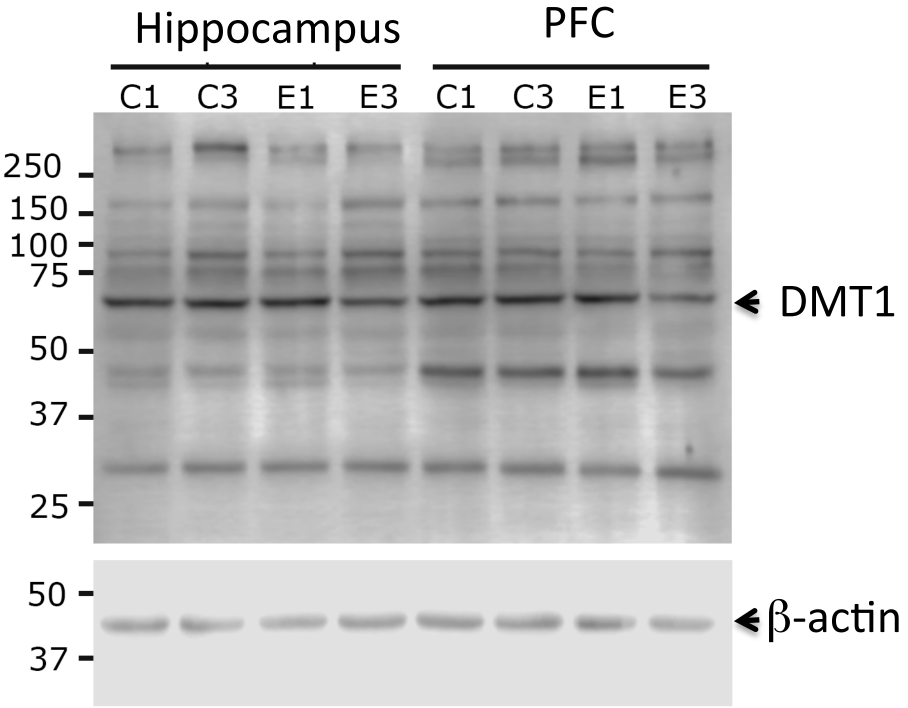


**BLOT6**

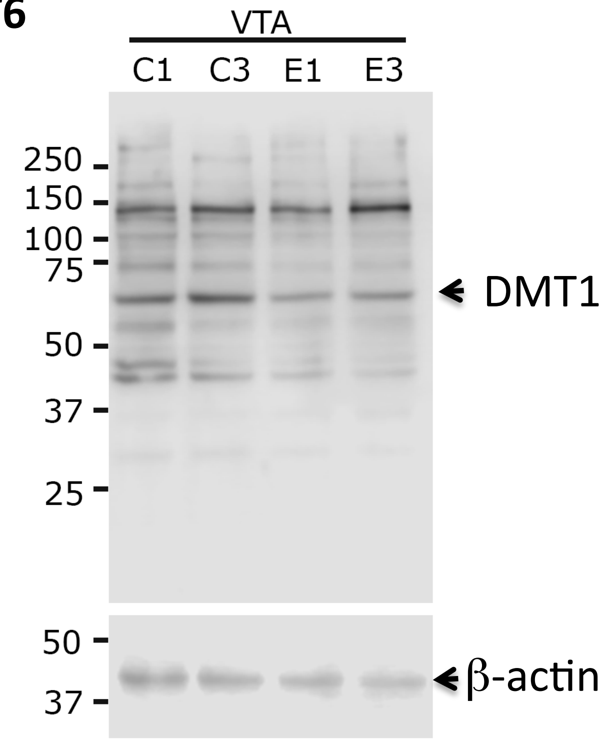


Hippocampus, control samples n=2, PAE samples ethanol n=2.
PFC, control samples n=2, PAE samples n=2.
VTA, control samples n=2, PAE samples n=2.

# WESTERN BLOT ANTI-TfR HIPPOCAMPUS

Control Samples (C), PAE Samples (E)

## BLOT 7

C1 E1 C2 E2 C3 E3 C4 E4

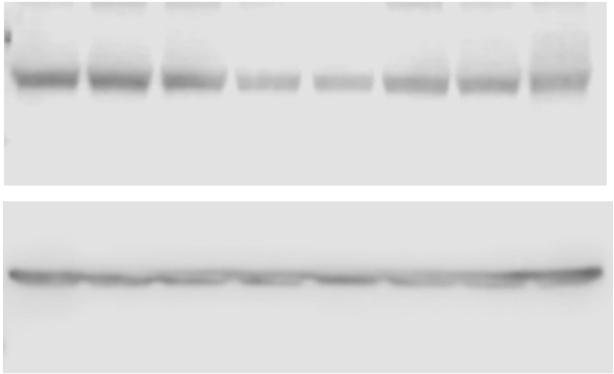

90KDa — TfR

42KDa — β-actin

## BLOT 8

C1 C2 E1 E2

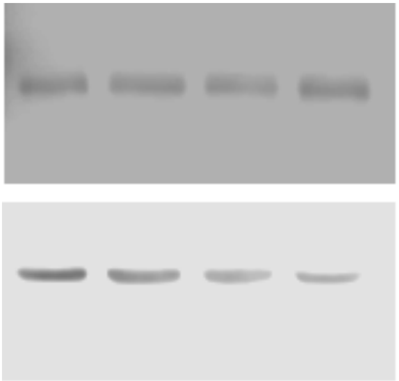

90KDa — TfR

42KDa — β-actin

Hippocampus, control samples n=6, PAE samples n=6
